# Lysine acetyltransferase 14 mediates TGF-β-induced fibrosis in ovarian endometrioma via co-operation with serum response factor

**DOI:** 10.1186/s12967-024-05243-2

**Published:** 2024-06-12

**Authors:** Yi Gong, Mian Liu, Qianqian Zhang, Jinjing Li, Hong Cai, Jing Ran, Linna Ma, Yanlin Ma, Song Quan

**Affiliations:** 1 Center for Reproductive Medicine, Department of Obstetrics and Gynecology, Nanfang Hospital, Southern Medical University, 1838 Guangzhou Avenue North, Guangzhou, Guangdong 510515 China; 2 Hainan Provincial Key Laboratory for Human Reproductive Medicine and Genetic Research, Hainan Provincial Clinical Research Center for Thalassemia, Key Laboratory of Reproductive Health Diseases Research and Translation, Ministry of Education, Department of Reproductive Medicine, Hainan Medical University, The First Affiliated Hospital of Hainan Medical University, Hainan Medical University, 54-1 LongHua road, Haikou, Hainan 570100 China; 3 Dongguan Maternal and Child Health Care Hospital, Postdoctoral Innovation Practice Base of Southern Medical University, Dongguan, 523001 China; 4 Department of Medical Genetics, School of Basic Medical Sciences, Southern Medical University, Guangzhou, 510515 China; 5 Department of Obstetrics and Gynecology, Shenzhen Hospital of Southern Medical University, Shenzhen, 518000 China; 6 Fujian Provincial Key Laboratory of Reproductive Health Research, Department of Obstetrics and Gynecology, School of Medicine, The First Affiliated Hospital of Xiamen University, Xiamen University, Xiamen, 361102 China

**Keywords:** KAT14, SRF, TGF-β, Endometrioma-associated fibrosis, Epigenetic modification

## Abstract

**Background:**

Fibrogenesis within ovarian endometrioma (endometrioma), mainly induced by transforming growth factor-β (TGF-β), is characterized by myofibroblast over-activation and excessive extracellular matrix (ECM) deposition, contributing to endometrioma-associated symptoms such as infertility by impairing ovarian reserve and oocyte quality. However, the precise molecular mechanisms that underpin the endometrioma- associated fibrosis progression induced by TGF-β remain poorly understood.

**Methods:**

The expression level of lysine acetyltransferase 14 (KAT14) was validated in endometrium biopsies from patients with endometrioma and healthy controls, and the transcription level of KAT14 was further confirmed by analyzing a published single-cell transcriptome (scRNA-seq) dataset of endometriosis. We used overexpression, knockout, and knockdown approaches in immortalized human endometrial stromal cells (HESCs) or human primary ectopic endometrial stromal cells (EcESCs) to determine the role of KAT14 in TGF-β-induced fibrosis. Furthermore, an adeno-associated virus (AAV) carrying *KAT14*-shRNA was used in an endometriosis mice model to assess the role of KAT14 in vivo.

**Results:**

KAT14 was upregulated in ectopic lesions from endometrioma patients and predominantly expressed in activated fibroblasts. In vitro studies showed that KAT14 overexpression significantly promoted a TGF-β-induced profibrotic response in endometrial stromal cells, while KAT14 silencing showed adverse effects that could be rescued by KAT14 re-enhancement. In vivo, Kat14 knockdown ameliorated fibrosis in the ectopic lesions of the endometriosis mouse model. Mechanistically, we showed that KAT14 directly interacted with serum response factor (SRF) to promote the expression of α-smooth muscle actin (α-SMA) by increasing histone H4 acetylation at promoter regions; this is necessary for TGF-β-induced ECM production and myofibroblast differentiation. In addition, the knockdown or pharmacological inhibition of SRF significantly attenuated KAT14-mediating profibrotic effects under TGF-β treatment. Notably, the KAT14/SRF complex was abundant in endometrioma samples and positively correlated with α-SMA expression, further supporting the key role of KAT14/SRF complex in the progression of endometrioma-associated fibrogenesis.

**Conclusion:**

Our results shed light on KAT14 as a key effector of TGF-β–induced ECM production and myofibroblast differentiation in EcESCs by promoting histone H4 acetylation via co-operating with SRF, representing a potential therapeutic target for endometrioma-associated fibrosis.

**Supplementary Information:**

The online version contains supplementary material available at 10.1186/s12967-024-05243-2.

## Background

Endometriosis is characterized by the presence of endometrial-like tissues outside the uterus, affecting approximately 10% of women of reproductive age worldwide [1, 2]. The clinical presentations of endometriosis exhibit significant heterogeneity and are associated with a range of painful symptoms, including chronic pelvic pain, dysmenorrhea, dyspareunia, dyschezia, and dysuria, along with infertility [3]. Endometriosis imposes a substantial economic burden, exceeding US$22 billion in the USA and £12.5 billion in the UK [4]. Previous studies have demonstrated that ovarian endometrioma (also known as endometriotic ovarian cyst), one of the main subtypes of endometriosis marked by lesions on the ovary forming large cystic structure, significantly contributes to endometriosis-associated symptoms [5, 6]. Emerging evidence has linked dysmenorrhea to endometrioma, and a growing body of research underscores the detrimental impact of endometrioma on ovarian reserve and oocyte quality, both of which are crucial components of female fertility [7, 8]. While these findings underscore the pathophysiological role of endometrioma in pain and infertility associated with endometriosis, the precise mechanisms underlying these symptoms remain elusive.

Endometrioma-associated fibrosis, a key pathological hallmark of endometrioma, is characterized by the overactivation of myofibroblasts and excessive deposition of extracellular matrix (ECM) tissues. Accumulating evidence has revealed that endometrioma-associated fibrosis contributes to scar formation and impairs the surrounding ovarian cortex tissues, leading to reduced ovarian reserve and dysmenorrhea [9]. Thus, a call has been made for increased attention to endometrioma-associated fibrogenesis in the counseling and management of patients with endometrioma due to its iterative and time-dependent nature, which holds preventive and interventional implications.

Numerous studies have highlighted ectopic endometrial stromal cells (EcESCs) as key cellular players in endometrioma-associated fibrosis [10]. EcESCs, which are precursors to myofibroblasts in endometrioma lesions that undergo activation and differentiation into myofibroblasts upon stimulation by transforming growth factor-β (TGF-β), a prominent activator that is abundant in endometrioma lesions. This differentiation is characterized by phenotypic changes, such as increased α-smooth muscle actin (α-SMA) expression, the release of cytokines, and pathological ECM production. Therapies targeting TGF-β-induced differentiation of EcESCs into myofibroblasts have emerged as compelling and clinically relevant strategies to address endometrioma-associated fibrosis progression and its associated symptoms, representing a clear priority in addressing the unmet medical needs of patients suffering from this disease.

Histone acetylation, modulated by histone acetyltransferases (HATs) and histone deacetylases (HDACs), is a post-translational modification crucial for cellular functions, facilitating DNA accessibility to the transcription machinery and promoting gene transcription [11]. Indeed, aberrant histone acetylation is closely related to the occurrence and development of various diseases, including metabolism disorder [12], cancer [13], and fibrotic disease [14]. Previous studies have implicated HDACs in the development of endometriosis lesions; indeed, prohibiting HDACs significantly attenuates the progress of endometriosis, suggesting that aberrant histone acetylation modifiers play a pathophysiological role in endometriosis development and may represent a potential therapeutic target for endometriosis [15–17]. However, the understanding of histone acetylation in endometrioma-associated fibrosis remains limited.

Lysine acetyltransferase 14 (KAT14, also known as CSRP2BP) is a subunit of the Ada2a-containing (ATAC) complex, which possesses HAT activity. We have previously reported the significant role of KAT14 in increasing α-SMA expression in smooth muscle cells by directly interacting with serum response factor (SRF) [18], a key transcription factor responsible for regulating fibrosis progression [19]. Given the role of KAT14 role in promoting α-SMA expression, we hypothesized that TGF-β-mediated myofibroblast activation in the endometrioma could be facilitated by KAT14. In this study, we identified and functionally characterized KAT14, which had high expression in endometrioma lesions, as a central regulator of TGF-β-induced pro-fibrotic effects within the progression of endometrioma-associated fibrogenesis. Through various experiments, we demonstrated how KAT14 influences TGF-β-induced endometrial stromal cell activation and pro-fibrotic responses by forming a complex with SRF, which directly. Our findings highlight the therapeutic potential of targeting the KAT14/SRF complex to limit excessive fibrosis progression in endometrioma.

## Methods

### Human samples

A cohort of 31 female patients aged 23 to 40 years who underwent laparoscopy for ovarian endometrioma (OE) were recruited from Nanfang Hospital, Southern Medical University, and from the First Affiliated Hospital of Hainan Medical University. Additionally, a control group of 23 women without endometriosis who were undergoing laparoscopic or hysteroscopy for other benign gynecological diseases, such as tubal disease or uterine myomas, was established. All participants had regular menstrual cycles, with their menstrual history duly confirmed. The diagnosis of ovarian endometrioma was substantiated through meticulous histological examination. Preceding surgical interventions, patients were required to refrain from hormonal treatments and intrauterine contraception for a minimum of 6 months. OE samples comprised tissues from ovarian endometriotic cysts (*n* = 31) and their eutopic proliferative phase endometrium (*n* = 31), while the control group comprised tissues from the eutopic proliferative phase endometrium (*n* = 23). All participants were in the proliferative phase of their menstrual cycles. Samples from these participants were divided into two portions. One portion was immediately collected to isolate primary human EcESCs, eutopic endometrial stromal cells (EuESCs), and normal endometrial stromal cells (NESCs) for a series of in vitro experiments, including RNA isolation, protein extraction, lentivirus infection, collagen gel contract, and migration assays. The other portion was fixed for immunofluorescence and immunohistochemistry experiments. All participants signed informed consent for collection of their endometrial and endometriotic tissues. The basic information of all participants is shown in Additional file 1: Tables S1, S2.

### Analysis of published single cell RNA-seq data

The single-cell transcriptomic data used in this study were retrieved from the NCBI Gene Expression Omnibus with the GEO series number GSE213216 [20]. To ensure data quality, low-quality cells were excluded based on the following criteria: (1) < 200 genes; or (2) > 20% unique molecular identifiers (UMIs) derived from the mitochondrial genome. Doublets were identified and removed using the R package, DoubletFinder (v.2.0.3), under the default settings. The batch effect across different samples was removed using the R package, harmony (v.0.1.1). The standard procedures of filtering, variable gene selection, dimensionality reduction and clustering were performed using the single cell RNA seq analysis R package Seurat (v.4.3.0). Mesenchymal cells were defined by the expression of cell type-associated genes: *DCN, COL11A2, FAP, PDGFRA, COL11A1, COL1A1*, and *PDGFRB.* Mesenchymal cell types were further sub-clustered to further decode the mesenchymal landscape in endometriosis. Scaling, principal component analysis, and clustering were performed as described above. Subsequently, the subclusters were annotated based on reported cell-specific marker genes [20]. Differential expression of the various clusters was also analyzed using Seurat, employing the Wilcoxon rank sum test.

**Fig. 1:**
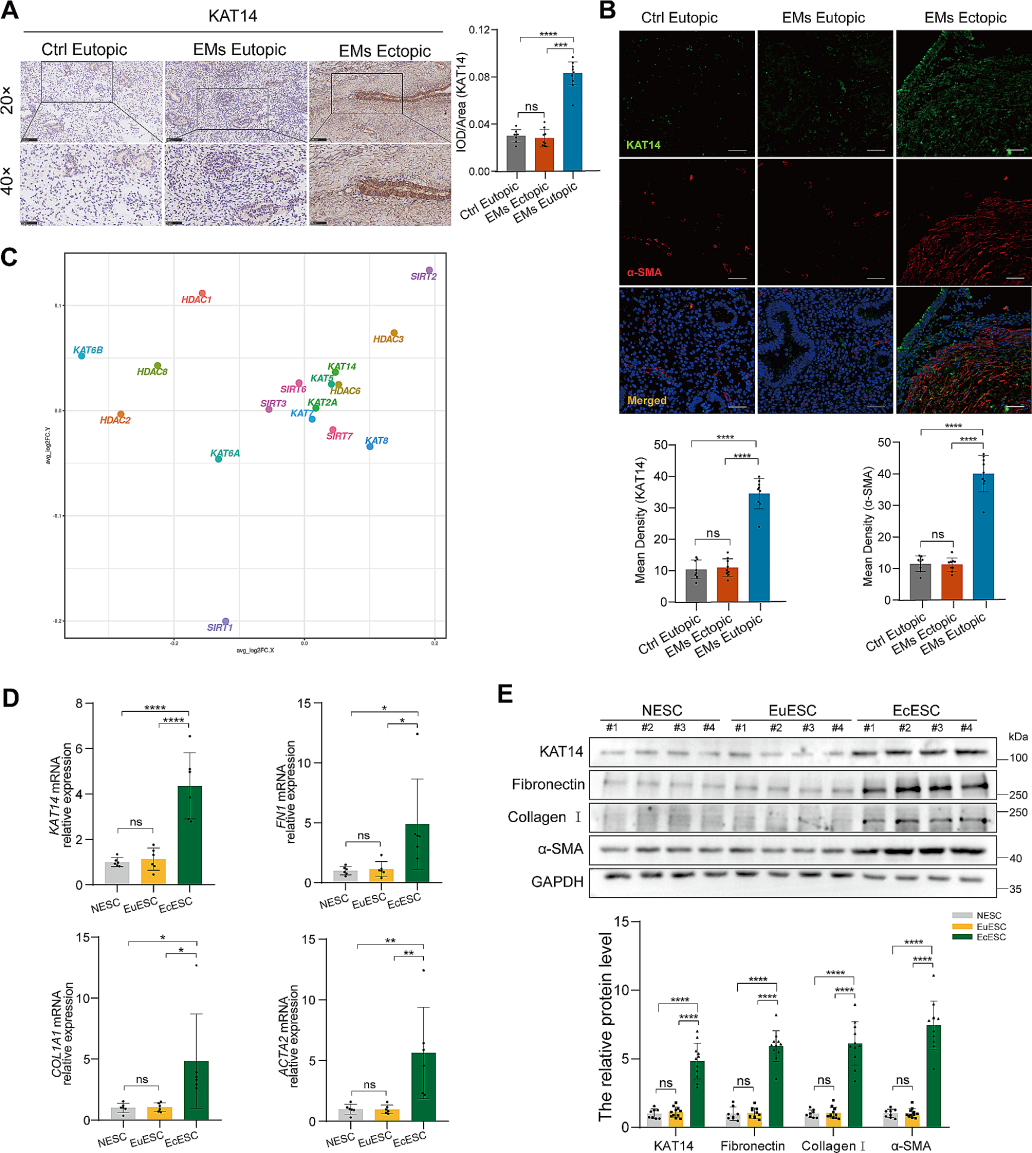
KAT14 was upregulated in human ovarian endometrioma lesions and primary human EcESCs. (**A)** Representative immunohistochemical staining for KAT14 in human normal endometrium tissue (*n* = 8), eutopic endometrium (*n* = 9), and ectopic lesions (*n* = 9) from human ovarian endometrioma. Scale bar: 100 μm (upper panel), scale bar: 50 μm (lower panel). (**B)** Double labeling immunofluorescence analysis showing the expression and distribution of KAT14 and α-SMA within normal endometrial tissues (*n* = 8), eutopic endometrium tissues (*n* = 9), and ectopic lesions (*n* = 9) from patients with ovarian endometrioma. Scale bar: 50 μm. (**C)** Single-cell differential expression analyses of histone acetylation genes in endometriomas, endometriosis, and eutopic endometrium. Each point (hollow circle) represents a single histone acetylation gene. The X-axis displays the average logarithm of fold change (logFC) values for genes measured in activated fibroblasts derived from endometrioma and endometriosis compared to those from the eutopic endometrium. The Y-axis represents the average logFC values for genes measured in all activated fibroblasts from endometrioma and endometriosis in relation to other cell populations from the same conditions. (**D**) qRT-PCR analysis of *KAT14*, *FN1*, *COL1A1*, and *ACTA2* transcripts in primary EcESCs (*n* = 6) compared to NESCs (*n* = 6) and EuESCs (*n* = 6). Relative quantification of gene expression was calculated using the 2−∆∆Ct method and normalized to *GAPDH* as the internal control. (**E**) Western blot was used to measure the protein level of KAT14, Fibronectin1, Collagen I, and α-SMA in primary EcESCs (*n* = 11) compared to NESCs (*n* = 8) and EuESCs (*n* = 11). Data are representative of three or more independent experimental replicates. For all panels, data are presented as the mean ± SD. *P*-values were determined by one-way ANOVA. *P < 0.05, **P < 0.01, ***P < 0.001, ****P < 0.0001, ns: Not significant. EMs: endometriomas, EcESCs: ectopic endometrial stromal cells, EuESCs: eutopic endometrial stromal cells, NESCs: normal endometrial stromal cells

**Fig. 2:**
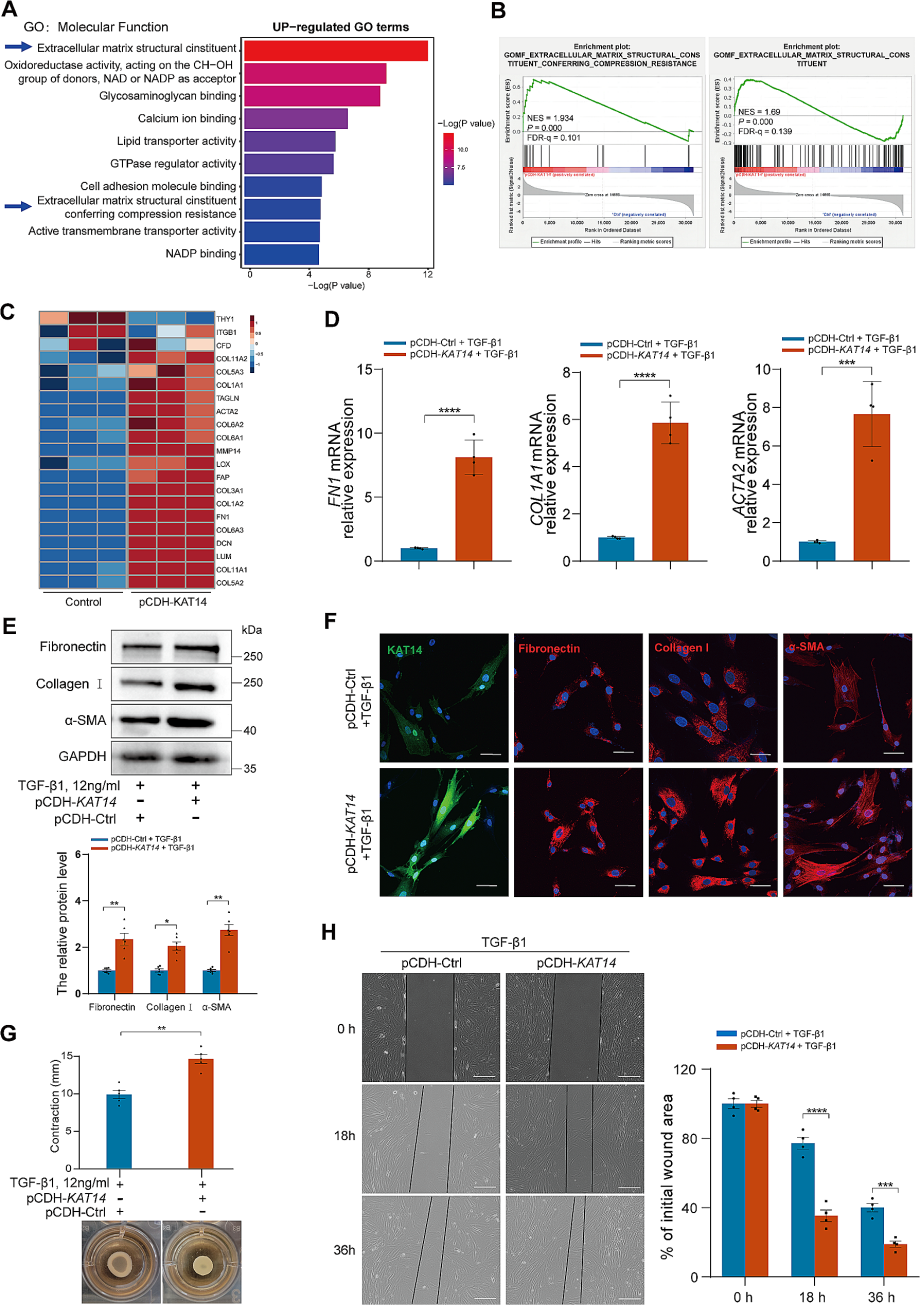
*KAT14* overexpression exacerbated TGF-β1-induced fibrogenesis in immortalized HESCs. (**A)** Bar graph showing the enrichment analysis of upregulated genes after KAT14 overexpression in terms of GO molecular function. (**B)** Gene set enrichment analysis (GSEA) of DEGs induced by KAT14 overexpression compared to the negative control showing significant enrichment in gene sets associated with extracellular matrix structural constituents and ECM component pathways. Normalized enrichment score (NES), false discovery rate (FDR), and *P-value*s are shown. (**C)** Heatmap profile of DEGs associated with fibroblast activation and ECM remodeling in KAT14-overexpressed cells compared to control cells based on RNA-Seq (*n* = 3). (**D)** qRT-PCR analysis of the relative mRNA expression of *FN1*, *COL1A1*, and *ACTA2* in HESCs infected with the indicated lentiviruses harboring *KAT14* expression vector (pCDH-*KAT14*) or empty vector control (pCDH-Ctrl) treated with TGF-β1 for 24 h. Relative quantification of gene expression was calculated using the 2^−∆∆Ct^ method and normalized to *GAPDH* as the internal control. (**E)** Western blots measuring the protein level of fibronectin, collagen I, and α-SMA in HESCs infected with pCDH-*KAT14* or pCDH-Ctrl lentiviruses under TGF-β1 stimulation for 48 h. (**F)** Immunofluorescence staining showing the expression of KAT14, α-SMA, and the ECM molecules fibronectin and collagen I in HESCs infected with pCDH-*KAT14* or pCDH-Ctrl lentiviruses under TGF-β1 stimulation for 48 h. Scale bar: 50 μm. (**G)** Collagen gel contractility assay showing the cell contraction capacity of HESCs infected with pCDH-*KAT14* or pCDH-Ctrl lentiviruses under TGF-β1 stimulation for 24 h. The degree of collagen gel contraction was determined as the difference between the diameters of the well and the released gels. (**H)** Wound healing assay showing the migration ability of HESCs infected with lentiviral vector containing pCDH-*KAT14* or pCDH-Ctrl treated with TGF-β1 for 0, 18, and 36 h. Wound healing was assessed by calculating the area in µm^2^ between the lesion edges. Scale bar: 200 μm. TGF-β1 was used at a concentration of 12 ng/ml. Data are representative of three or more independent experimental replicates. For all panels, data are presented as the mean ± SD. *P*-values were determined by Student’s t-test in panels (**D**, **E**, **G**, **H**). *P **<** 0.05, **P < 0.01, ***P < 0.001, ****P < 0.0001. HESC: human endometrial stromal cell, GO: gene ontology, DEGs: differentially expressed genes, Ctrl: control

**Fig. 3:**
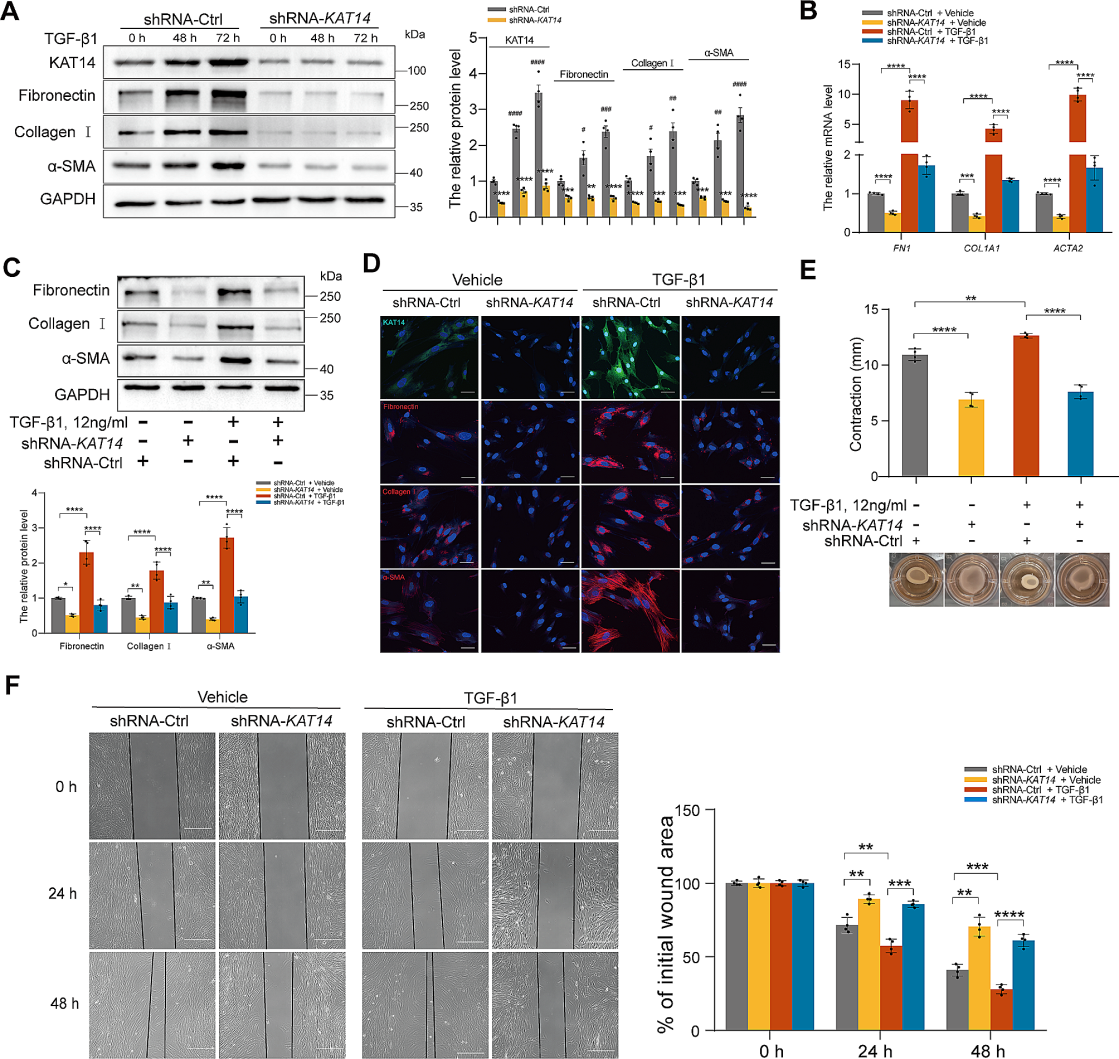
Knockdown of *KAT14* attenuated TGF-β1-induced fibrogenesis in primary human EcESCs. (**A)** Western blot showing the protein levels of fibronectin, collagen 1, and α-SMA in primary EcESCs infected with shRNA-*KAT14* or shRNA-Ctrl lentiviruses treated with TGF-β1 or vehicle for 0, 48, and 72 h. **P < 0.01, ***P < 0.001, and ****P < 0.0001 shRNA-*KAT14* group versus shRNA-Ctrl group; ^#^*P* < 0.05, ^##^*P* < 0.01, ^###^*P* < 0.001, ^####^*P* < 0.0001 shRNA-Ctrl group at 48–72 h versus shRNA-Ctrl at 0 h. **(B)** qRT-PCR analysis of the relative mRNA expression of *FN1*, *COL1A1*, and *ACTA2* in primary EcESCs infected with shRNA-*KAT14* or shRNA-Ctrl lentiviruses treated with TGF-β1 or vehicle for 24 h. Relative quantification of gene expression was calculated using the 2^−∆∆Ct^ method and normalized to *GAPDH* as the internal control. (**C)** Western blots were used to measure the protein level of fibronectin, collagen I, and α-SMA in primary EcESCs infected with shRNA-*KAT14* or shRNA-Ctrl lentiviruses stimulated by TGF-β1 or vehicle for 24 h. (**D)** Immunofluorescence staining showing the expression of KAT14, HESC activation marker, α-SMA, and the ECM molecules fibronectin and collagen I in primary EcESCs infected with shRNA-*KAT14* or shRNA-Ctrl lentiviruses stimulated by TGF-β1 or vehicle for 48 h. Scale bar: 50 μm. (**E)** Collagen gel contractility assay showing the cell contraction capacity of primary EcESCs transfected with shRNA-*KAT14* or shRNA-Ctrl lentiviruses stimulated by TGF-β1 or vehicle for 24 h. The degree of collagen gel contraction was determined as the difference between the diameters of the well and the released gels. (**F)** Wound healing assay showing the migration ability of primary EcESCs infected with shRNA-*KAT14* or shRNA-Ctrl lentiviruses stimulated by TGF-β1 or vehicle for 0, 24, and 48 h. Wound healing was assessed by calculating the area in µm^2^ between the lesion edges. Scale bar: 200 μm. The concentration of TGF-β1 used for panels (**A**–**F**) was 12 ng/ml. Data are representative of three or more independent experimental replicates. For all panels, data are presented as the mean ± SD. *P*-values were determined by Student’s t-test in panel (**A**) and by one-way ANOVA in panels (**A**–**C**, **E**, **F**). *P < 0.05, **P < 0.01, ***P < 0.001, ****P < 0.0001. EcESCs: ectopic endometrial stromal cells, Ctrl: control

### Animal study

**Fig. 4:**
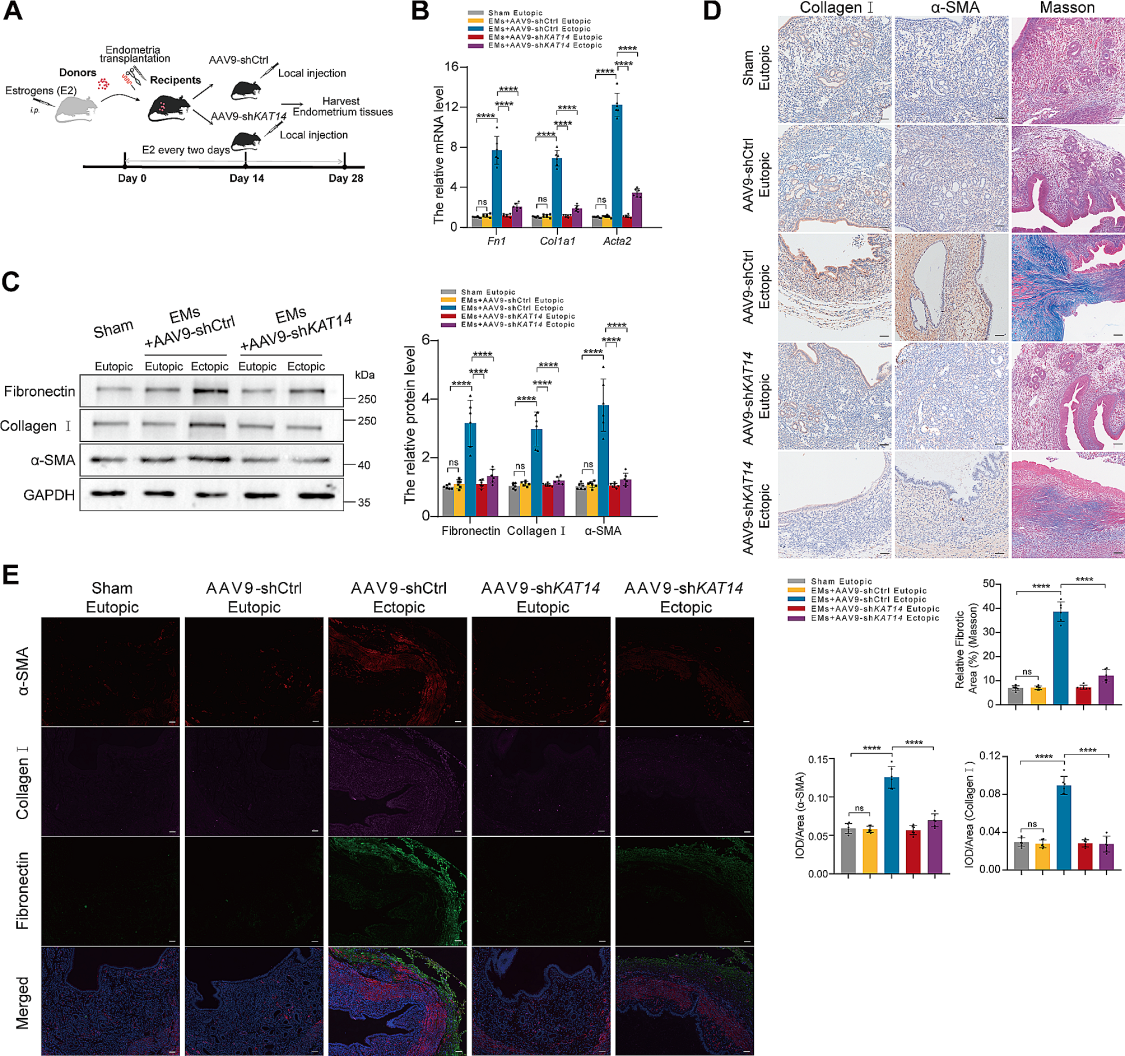
*KAT14* knockdown ameliorates endometriosis-associated fibrosis in vivo. (**A)** Schematic diagram of the in vivo experiments. (**B)** qRT-PCR analysis of *Fn1*, *Col1a1*, and *Acta2* expression in eutopic endometrium tissues from sham control, ectopic endometrial-like lesions, and eutopic endometrium tissues from endometriosis mice locally injected with AAV9-sh*KAT14* or its negative control (AAV9-shCtrl). Relative quantification of gene expression was calculated using the 2^−∆∆Ct^ method and normalized to *Gapdh* as the internal control; *n* = 6 mice per group. (**C)** Western blotting analysis of fibronectin, collagen I, and α-SMA expression in eutopic endometrium tissues from sham control, ectopic endometrial-like lesions, and eutopic endometrium tissues from endometriosis mice injected with AAV9-shCtrl or AAV9-sh*KAT14*; *n* = 6 mice per group. (**D)** Representative images of collagen I and α-SMA immunohistochemistry and Masson’s staining in eutopic endometrium tissues from sham control, ectopic endometrial-like lesions, and eutopic endometrium tissues from AAV9-shCtrl injected or AAV9-sh*KAT14* injected endometriosis mice; *n* = 6 mice per group, Scale bars: 50 μm. (**E)** Representative triple immunofluorescence staining images of collagen I, α-SMA, and fibronectin in eutopic endometrium tissues from sham control, eutopic endometrium tissues from sham control, ectopic endometrial-like lesions, and eutopic endometrium tissues from AAV9-shCtrl injected or AAV9-sh*KAT14* injected endometriosis mice; *n* = 6 mice per group, Scale bars: 50 μm. Data are shown as the mean ± SD. *P*-values were determined by one-way ANOVA. **P < 0.01, ***P < 0.001, ****P < 0.0001, ns: Not significant, AAV: adeno-associated virus

Thirty 6-week-old female C57BL/6 mice (RRID: IMSR_JAX:000664) were purchased from ZHBY Biotech Co., Ltd (Nanchang, China). Adult female mice (6 weeks) underwent a 1-week quarantine period and were subsequently employed to establish experimental models under specific pathogen-free conditions. An established mouse-mouse intraperitoneal transplantation model, as outlined in prior studies [21] with modifications, was used to generate the endometriosis (EMs) model. To eliminate potential variation, the endometrial tissue fragments from two donor mice were mixed and then divided into four parts, each stitched to two mice drawn from one of the two experimental groups on day 0, as per the experimental design. The sham group was established using the same steps as the endometriosis surgeries, excluding the suturing of any tissue to the abdominal wall. Eight mice were randomly selected as donors for this experiment, and EM mouse models were successfully established in 14 recipient mice. For KAT14 knockdown assays, EM mice were randomized into two groups (*n* = 6 per group): (1) EM mice treated with AAV9 carrying shRNA for mouse *KAT14* (AAV9-sh*KAT14*); and (2) EM mice treated with the negative control (AAV9-shCtrl). Both groups received local injections of AAV9 carrying sh*KAT14* or its negative control shCtrl on day 14 after endometrial tissue implantation. Mice were euthanized on day 28, and uterine and ectopic lesions were collected for subsequent studies. The detailed animal experiment process is shown in Fig. 4A.

### Cell lines and cell culture

The immortalized human endometrial stromal cell line, generously provided by Prof. Haibin Wang (Xiamen University), underwent cell immunofluorescence staining to determine the purity of the human endometrial stromal cells (HESCs) using pan-cytokeratin (#26411-1-AP, Proteintech, RRID: AB_2880505), CD10 (#23898-1-AP, Proteintech, RRID: AB_2879354), and vimentin (#60330-1-Ig, Proteintech, RRID: AB_2881439) (Additional file 1: Fig. S7A), as previously described [22]. The HESCs were cultured according to established protocols [23] involving a maintenance medium of DMEM/F-12 containing 10% (v/v) charcoal-stripped fetal bovine serum (CS-FBS, #04-201-1 A, BIOLOGICAL INDUSTRIES), 3.1 g/L glucose (#D823520, Macklin), and 1 mM sodium pyruvate (#S817535, Macklin) supplemented with 1% penicillin–streptomycin, 1.5 g/L sodium bicarbonate (#S818079, Macklin), 500 ng/ml puromycin (#P8230, Solarbio), and 1% insulin–transferrin–selenium (#51,500,056, Thermo Fisher) at 37 °C and 5% CO_2_. Wild-type HESCs or established stable *KAT14*-KO HESCs were stimulated with 12 ng/ml TGF-β1 (#100 − 21, PEPROTECH) or vehicle (PBS) and harvested for subsequent experiments.

Primary endometrial stromal cells (ESCs) were isolated and cultured as previously described [22]. In brief, the proliferative phase endometrial and endometriotic tissues were washed with sterile D-Hanks Balanced Salt Solution (HBSS, #H1045, Solarbio) and 1% penicillin–streptomycin (#15,140,122, Gibco) and then carefully dissected and minced into small pieces, approximately 1 mm^3^ in size. The minced tissues were incubated in HBSS containing type IV collagenase (4%) (#17,104,019, Gibco) in a shaking incubator for 90 min at 37 °C. The undigested tissue was removed by centrifugation at 200 g for 3 min. The cell suspension was passed through a graduated series of nylon mesh cell strainers (70 μm and 40 μm) (#352,350, BD; #352,340, BD) and then washed three times by hypotonic lysis (#R1010, Solarbio) to remove the erythrocytes. Finally, the ESC pellets were resuspended in a maintenance medium composed of DMEM/F-12 supplemented with 10% fetal bovine serum (FBS; #10270-106, Gibco) and 1% penicillin–streptomycin, plated on a 60-mm cell culture dish (#430,166, Corning), and incubated at 37 °C in 95% air/5% CO_2_. Forty-eight hours after cells seeding, the original medium was replaced and the unattached epithelial cells were removed by rinsing with phosphate buffered saline (PBS) (#C10010500BT, Gibco). The ESCs were passaged and used before the fourth passage for experiments. Next, cell immunofluorescence staining was conducted to determine the purity of the isolated ESCs using antibodies for human pan-cytokeratin (#26411-1-AP, Proteintech, RRID: AB_2880505), CD10 (#23898-1-AP, Proteintech, RRID: AB_2879354), and Vimentin (#60330-1-Ig, Proteintech, RRID: AB_2881439 (Additional file 1: Fig. S6A), as previously described [22]. In some experiments, primary ESCs were cultured with either TGF-β1 or the SRF inhibitor CCG-1423 (#SML0987, Sigma) for 24–48 h, with CCG-1423 dissolved in DMSO (#D2650, Sigma), while the same volume of DMSO was added to the controls.

Human embryonic kidney 293T cells (HEK 293T) (RRID: CVCL_0063) were purchased from IGE Biotechnology Co. Ltd. (Guangzhou, China) and cultured at 37 °C and 5% CO_2_, with regular passaging. The HEK 293T cell line underwent short tandem repeat profiling for identification and was confirmed to be free of mycoplasma contamination before the experiments.

### RNA sequencing (RNA-seq) and data analysis

HESCs were cultured in DMEM/F-12 medium and transfected with pCDH-*KAT14* or pCDH-control lentiviruses. After treatment, total RNA was extracted using TRIzol reagent (#15596-018, Invitrogen) following the standard protocol. Then, the RNA quality and quantity were assessed using a NanoDrop One/OneC Microvolume UV-Vis spectrophotometer (Thermo Fisher Scientific) and Qubit 2.0 Fluorometer (Invitrogen), the integrity of RNA was confirmed using an Agilent 4200 TapeStation System. The cDNA libraries were generated by HaploX Biotechnology (Jiangxi, China). Specifically, the transcriptome library for sequencing was generated using the NEBNext Ultra™ II mRNA Library Prep Kit for Illumina kit (#E7770L, NEB) according to the manufacturer’s specifications. Furthermore, the library fragments were qualified/quantified using TapeStation4200 (Agilent). The RNA-seq libraries were sequenced on the Illumina PE150 system. Raw data in fastq format were processed with fastp. Clean data were obtained after quality control, adapter trimming, quality filtering, and per-read quality cutting. All of the downstream analyses were based on the clean data with high quality. Then, paired-end clean reads were aligned to the reference genome using HISAT2 software v2.1.0 [24]. Differential gene expression (|log2(FoldChange)| > 1 and adjusted P-value < 0.05) analysis of two groups was performed using the DESeq R package (1.18.1) [25]. Gene Ontology (GO) enrichment analysis and visualization of differentially expressed genes was implemented by the ClusterProfiler R package [26]. Gene set enrichment analysis (GSEA) was performed using GSEA software v4.1.0 [27].

### Lentiviral vectors and stable cell line construction

The total RNA from immortalized human HESCs was isolated using TRIzol following the manufacturer’s protocol. The full length coding sequence of human wild-type *KAT14* (NM_001392973.1) cDNA was synthesized by reverse transcription-PCR (RT-PCR) using the HiScript II 1st Strand cDNA Synthesis Kit (#R211-01, Vazyme). To generate plasmids for mammalian expression of KAT14, *KAT14* cDNA fused with an HA tag at the N-terminal region was subcloned into the pCDH-CMV-MCS-EF1-puro plasmid (IGE Biotechnology Co. Ltd., Guangzhou, China). All constructs were confirmed by direct sequencing. The shRNA (short hairpin RNA) targeting *KAT14* was designed from the website https://www.sigmaaldrich.cn/CN/zh. After annealing, the double-stranded oligonucleotides were subcloned into the EcoRI and AgeI restriction sites of pLKO.1 vectors (#8453, Addgene, RRID: Addgene_8453).

All lentiviruses used in this study (viral particles containing plasmids that express *KAT14* or the shRNA-*KAT14*) were generated in HEK 293T cells via polyethyleneimine linear (#40816ES02, YEASEN) transfection, as previously described [28]. For lentivirus infection, target cells were seeded in a 24-well plate (#3513, Corning) (4 × 10^4^ cells per well), cultured for 24 h, and then incubated with viral particles. To establish stable *KAT14*-knockdown (KD) cell lines, primary human EcESCs were transfected with shRNA-*KAT14* or shRNA-control lentiviruses. For the KAT14 rescue experiment, *KAT14* overexpression (pCDH-*KAT14*) lentiviruses and the empty vector were transfected into the previously established stable *KAT14*-knockout (KO) immortalized HESCs or the established stable *KAT14*-KD EcESCs. Cells infected with lentivirus were selected with Puromycin (#P8230, Solarbio) (2.5 µg/ml for immortalized HESCs, 4 µg/ml for EcESCs) to eliminate non-infected cells 72 h after infection, yielding stably transfected cell lines for subsequent experiments. Detailed information on these shRNA target sequences is provided in Additional file 1: Table S3.

### Clustered regularly interspaced short palindromic repeats (CRISPR)-Cas9 *KAT14* knockout

For genome editing in immortalized HESCs, single guide RNA (sgRNA) targeting *KAT14* was designed using an optimized CRISPR online tool (http://crispr.mit.edu/), as previously described [29]. Specific *KAT14* sgRNA oligos were annealed and cloned into lentiCRISPR v2 (#52,961, Addgene, RRID: Addgene_52961), encoding the Cas9 protein. Lentivirus packaging was performed as described above. Immortalized HESCs were seeded in a 24-well plate (4 × 10^4^ cells per well) and incubated with virus supernatant. Seventy-two hours after infection, immortalized HESCs were screened with 2.5 µg/ml puromycin for 1 week to select stably transfected cell lines for further experiments. The puromycin-selected cells were diluted into 96-well plates (#3599, Corning) at densities of 15–20 cells/ml to obtain individual cells for further single cell clone establishment. For each clonal population, 50 to 100 cells were harvested to isolate genomic DNA 12–14 days after cell seeding and subjected to direct PCR to amplify fragments containing the editing site. The knockout efficiency of cells was validated by sequencing the PCR amplification products and western blotting. Detailed information of the sgRNA target sequences are shown in Additional file 1: Table S3.

### Small interfering RNA (siRNA) transfection

The established stable *KAT14*-KD primary human EcESCs were seeded onto 100-mm cell culture dishes (#430,167, Corning) for chromatin immunoprecipitation, 6-well plates (#3516, Corning) for western blot assay, or 24-well plates for luciferase assay and gel contraction assays. The cells were transfected with siRNA-*SRF* and the nonsense mutation negative control (siRNA-ctrl) (IGE Biotechnology Co. Ltd., Guangzhou, China) at approximately 60% confluence. siRNA transfections were performed in serum-free OptiMEM (#31,985,062, Gibco) using Lipofectamine 3000 (#L3000008, Invitrogen) according to the manufacturer’s protocol. After 36 h of incubation, the culture media was refreshed and cells were stimulated with 12 ng/ml TGF-β1 or PBS (#C10010500BT, Gibco) for 24 h before conducting subsequent experiments. Detailed information on the siRNA target sequences are shown in Additional file 1: Table S3.

### RNA isolation, cDNA synthesis, and quantitative real-time PCR (qRT-PCR)

Cells and endometrial tissues were washed three times with PBS and then placed in TRIzol (#15596-018, Invitrogen) to isolate the total RNA following the manufacturer’s protocol. The cDNA was synthesized by reverse transcription-PCR with the PrimeScript RT reagent Kit (#RR047A, Takara). The primer sequences used in these experiments are shown in Additional file 1: Table S4. Amplification was performed in a LightCycler 96 system (Roche, USA) using SYBR GreenTM Master Mix (#RR820A, Takara). The optimal concentration of each primer was determined to ensure high amplification efficiency and specificity in the subsequent qRT-PCR assay. All reactions were performed at least in triplicate. Relative quantification of gene expression was calculated using the 2^−∆∆Ct^ method and normalized to *GAPDH* as the internal control.

### Collagen gel contraction assay

The pretreated wild-type or *KAT14*-KO immortalized HESCs, as well as the primary human EcESCs, were collected and suspended at a density of 2.0 × 10^5^ cells per 100 µl of medium in 400 µl collagen gel working solution before aliquoting into 24-well plates. The resulting mixture was allowed to gel for 1 h at 37 °C. After polymerization, 1 ml of DMEM/F-12 containing 10% CS-FBS was added on top of the gel lattice and incubated for 24 h. Subsequently, the polymerized gels were released by running a sterile pipette tip along the sides of the well before being cultured for another 24 h in DMEM medium containing 12 ng/ml TGF-β1 or vehicle (PBS) only. The surface area of the contracted gels at 0, 12, and 24 h was measured using Image-Pro Plus v. 6.0 software (Media Cybernetics, Inc., Silver Spring, MD, USA). Measurements of the diameter of each gel were recorded as the average values of the major and minor axes, as previously described [30]. The gel contraction was calculated as follows: diameters of wells – diameters of contracted gels. All reactions were performed at least in triplicate.

### Luciferase reporter assay

The wild-type form of the 3′UTR region of the *ACTA2* promoter containing the CArG box was subcloned into the pGL3 reporter vector (#E1751, Promega, RRID: Addgene_48743). For the luciferase assay, the pretreated primary human EcESCs were plated on 24-well plates and co-transfected with 0.5 µg of pGL3-α-SMA-pro-Luc or empty (pGL3-Basic) plasmids together with 0.05 mg of pRL Renilla luciferase reporter vector (#E2241, Promega, RRID: Addgene_44379) using Lipofectamine 3000 according to the manufacturer’s instructions. The cells were incubated with TGF-β1 or vehicle (PBS) for 24 h and harvested to investigate the effects of KAT14 on primary EcESC α-SMA expression. The luciferase activity was measured using a dual-luciferase reporter assay kit (#11402ES80, YEASEN) following the manufacturer’s protocol.

### In vitro cell migration assay

The migration capability of ESCs was assessed by wound healing assays. In the in vitro wound healing assay, cells were seeded at 2 × 10^5^ cells per well in a 6-well plate and stimulated with 12 ng/ml TGF-β1 or vehicle (PBS) for another 24 h at 37 °C. Subsequently, a scratch was created in the monolayered cell with a sterile pipette tip and then washed three times with PBS. The cells were cultured with FBS-free DMEM/F-12 medium at 37 °C in a humidified 5% CO_2_ atmosphere. The average width of each scratch was photographed under an inverted microscope and analyzed by Image-Pro Plus v. 6.0 software at 0, 24, and 48 h. Wound healing was measured by calculating the area in µm^2^ between the lesion edges. The experiment was performed four times.

### Antibodies

Detailed information of antibodies used in this study are listed in Additional file 1: Table S5.

### Western blot analysis

For total protein extraction, tissues and pretreated cells were washed with ice-cold PBS and incubated with whole-cell lysis buffer NP-40 (#P0013F, Beyotime) supplemented with protein inhibitor phenylmethylsulfonyl fluoride (PMSF, #ST505, Beyotime) and phosphatase inhibitor cocktail (#P5726, Sigma-Aldrich) for 30–50 min on ice and the supernatant was collected by centrifugation (13,000 rpm) at 4 °C for 20 min. Cytosolic and nuclear proteins were separated using a Nuclear and Cytoplasmic Protein Extraction Kit (#P0028, Beyotime). The total histones of primary human EcESCs were extracted using an EpiQuik Total Histone Extraction Kit (#OP-0006-100, Epigentek). Briefly, cells were harvested and washed with ice-cold PBS. The total histones were extracted after successive prelysis, lysis, and addition of balance buffers. The protein concentration of cell lysates was quantified using an Enhanced BCA Protein Assay Kit (#P0010, Beyotime) according to the manufacturer’s instructions. Equal amounts of proteins (20–50 µg/lane for total proteins; 40 µg/lane for cytosolic and nuclear proteins; 40 µg/lane for histone proteins, separately) were separated on 8–18% SDS-PAGE and transferred onto PVDF membranes (#ISEQ00010, Merck Millipore). The PVDF membranes were blocked with 5% skim milk (#D8340, Solarbio) for 2 h at room temperature and then incubated with the corresponding primary antibody, which was diluted in the range of 1:200 to 1:10000 (Additional file 1: Table S5) overnight at 4 °C with shaking. After washing with TBST, the membranes were incubated with HRP-conjugated secondary antibody for 1 h at room temperature. The protein signals were visualized by chemiluminescence (ECL) substrate (#34,577, Thermo Fisher Scientific) and detected using a Tanon 5200 imaging system (Tanon, Shanghai, China). The protein level was quantified by densitometry with Image-Pro Plus v. 6.0 software and normalized to the level of GAPDH, which served as the internal control to normalize the densitometric intensity. The information on the antibodies applied in this study is shown in Additional file 1: Table S5.

### Coimmunoprecipitation (CoIP)

The interaction between endogenous KAT14 and SRF was detected using a Co-IP assay. The primary human EcESCs in primary culture were harvested for immunoprecipitation assays according to standard protocols. Briefly, whole-cell protein was extracted and the cell lysates were centrifuged. The supernatant was collected and precleared by incubation with Protein A/G PLUS-Agarose (#sc-2003, Santa Cruz) and isotypic IgG, followed by incubation with an appropriate amount of specific primary antibody against KAT14 (#sc-398,475, Santa Cruz Biotechnology, RRID: AB_2936359) (4 µg), SRF (#16821-1-AP, Proteintech, RRID: AB_2194384) (2 µg), or normal rabbit IgG (#10284-1-AP, Proteintech, RRID: AB_2877729) (2 µg), separately. Then, resuspended Protein A/G PLUS-Agarose was added to each immunoprecipitation mixture. The beads were washed three times and centrifuged to obtain the indicated protein complexes, which were then resuspended in 1 x electrophoresis sample buffer and separated by SDS–PAGE. Proteins of interest were detected by a subsequent western blot assay with their corresponding antibodies.

### Chromatin immunoprecipitation (ChIP)

Enrichment of the target protein at the *ACTA2* promoter was analyzed by ChIP assays using a commercially available EZ-ChIP™ Chromatin Immunoprecipitation Kit (#17–371, Merck Millipore) based on the manufacturer’s instruction. Subsequently, the immunoprecipitated DNA fragments were analyzed by quantitative PCR to assess the enrichment of KAT14 at the promoters of *ACTA2* and *KPNA2*, which lacks potential KAT14 binding sites, was employed as a negative control. Additionally, the ChIP assay included PCR to assess the acetylation status of histone H4 at the ACTA2 proximal promoter. The ChIP-qPCR results are presented as enrichment relative to the input. The primers are summarized in Additional file 1: Table S4.

### Masson’s trichrome staining

Mouse model samples and human eutopic and ectopic endometrium tissues were fixed in 4% paraformaldehyde (#P1110, Solarbio) at room temperature for 2 h. After washing three times with PBS, the fixed tissues was embedded in paraffin and sectioned into 5-mm slices (5-µm thick). The endometrial morphology was visualized using a Masson’s trichrome staining kit (#G1345, Solarbio) according to the manufacturer’s instructions. The collagen fibers were stained light blue and quantification of fibrotic areas was undertaken by visualizing blue-stained areas using the Olympus IX53 microscope (Tokyo, Japan). Analysis of the endometrium involved two randomly chosen sections per sample, and the areas stained in blue, in proportion to the entire field were calculated using Image-Pro Plus v. 6.0 software.

### Immunohistochemistry (IHC) assay

For immunohistochemical staining of cells, approximately 4 × 10^4^ primary ESCs were plated on slides and fixed with 4% PFA. A series of consecutive sections and cell slides were immersed in xylene and ethanol for deparaffinization and hydration, respectively, and then subjected to antigen retrieval by boiling in citrate buffer. Subsequently, the tissue sections and cell slides were incubated overnight with the corresponding primary antibodies at 4°C, followed by immunohistochemical staining (#SAP-9100, ZSGB-BIO). Signal visualization was based on 3,3′-diaminobenzidine (DAB) (#G1212-200T, Servicebio), and cell nuclei were stained with hematoxylin. The images were taken with an Olympus IX53 microscope and analyzed using Image-Pro Plus v.6.0 software to determine the relative expression levels of objective proteins according to the integrated optical density (IOD) of the digital images. At least four randomly selected fields of view were analyzed per sample. The information on antibodies applied in this study is referred to Additional file 1: Table S5.

### Immunofluorescence assay

Cell slides and tissue sections were prepared following the aforementioned procedures. For staining, tissue sections and cell slides were permeabilized, and then incubated with the indicated primary antibodies (dilution ratios are provided in Additional file 1: Table S5) at 4 °C overnight. After three washes with 0.1% Tween 20 in PBS, the sections and cells were incubated with the corresponding secondary antibodies, including donkey anti-rabbit (conjugated with Alexa Fluor® 647), donkey anti-goat (conjugated with Alexa Fluor® 568), or goat anti-mouse (conjugated with Alexa Fluor® 488), at room temperature for 1 h. Following cell nuclear staining with an antifade mounting medium with DAPI (#P0131, Beyotime), the samples were mounted and immunofluorescence images were obtained with an Olympus FV3000 confocal microscope (Tokyo, Japan).

### Statistical analysis

Statistical analyses were performed using SPSS software version 24.0 (SPSS Inc, Chicago, USA) and GraphPad Prism 8.0.1 software (GraphPad, San Diego, California, USA). All data are shown as the mean ± standard deviation (SD) from at least three independent experiments. Two-tailed Student’s t-tests and the Mann–Whitney U test were employed to compare the quantitative variables between two groups. For experiments involving at least three groups, one-way analysis of variance (ANOVA) with Tukey’s multiple comparisons test was used. All sample measurements were performed in a blinded manner. *P*-values < 0.05 were considered to indicate statistical significance.

## Results

### KAT14 is upregulated in ovarian endometrioma

Although fibrosis is the main pathological feature of ovarian endometrioma, understanding of its underlying mechanism remains limited [5, 31]. Multiple studies have established the involvement of abnormal histone acetylation in the initiation and progression of endometriosis [15, 32, 33]. In this study, we investigated the protein level of KAT14, an HAT known to drive the expression of smooth muscle genes such as α-SMA, in endometrioma lesions. The presence of endometrioma lesions was histologically confirmed by IHC staining for useful immunohistochemical marker of endometriosis such as estrogen receptor (ER), progesterone receptor (PR), and CD10 [34–36] (Additional file 1: Fig. S1). Subsequently, we performed IHC staining to assess the expression of KAT14 in endometrioma lesions and the results revealed a significant increase in the intensity of KAT14 in sections from endometrioma lesions when compared to sections from the eutopic endometrium of patients with endometrioma and healthy controls (Fig. 1A). Immunofluorescence staining showed increased expression of KAT14 and α-SMA in endometrioma lesions, and the distribution of KAT14 was consistent with that of α-SMA (Fig. 1B), suggesting a correlation between KAT14 expression and fibrogenesis in endometrioma. We conducted a comprehensive analysis of the recently published single-cell transcriptomes (scRNA-seq) dataset [20], encompassing endometriomas, endometriosis, and eutopic endometrium (Fig. 1C). The results indicated a significant increase in the expression of *HDAC3, HDAC6*, *KAT2A*, *KAT5*, *KAT14*, and *SIRT2* in activated fibroblasts derived from endometrioma and endometriosis compared to those from the eutopic endometrium, suggesting the involvement of these genes in the development of endometriosis fibrosis. Intriguingly, further functional experiments revealed that only KAT14 exhibited a significant elevation at both the protein and transcription levels in endometriosis ectopic tissues (Additional file 1: Fig. S2A, B), which is consistent with our previous results and suggests a crucial role for KAT14 in endometrioma-associated fibrogenesis. To further elucidate the contribution of KAT14 in endometrioma-associated fibroblast activation, we examined the expression level of KAT14 and fibrotic proteins in primary human EuESCs, EcESCs collected from patients with endometrioma, and primary NESCs collected from healthy controls. Quantitative reverse transcription PCR (qRT-PCR) revealed a significant upregulation of *KAT14* mRNA, as well as typical fibrosis-associated molecules such as *FN1* (fibronectin encode gene), *COL1A1* (collagen I encode gene), and *ACTA2* (α-SMA encode gene), in EcESCs compared to human NESCs and EuESCs (Fig. 1D). These findings were consistent with the immunoblotting results (Fig. 1E). Taken together, these results suggest that KAT14 is a key HAT involved in the progression of endometrioma-associated fibrogenesis.

### Overexpression of KAT14 promotes TGF-β1-induced fibrogenesis in immortalized human endometrial stromal cells (HESCs)

TGF-β1 is one of the key cytokines which significantly increased in endometriosis and recognized as the main driver of fibrogenesis, primarily through its role in promoting myofibroblast activation and the synthesis of extracellular molecules in endometriosis [37–39]. In current study, we found the expression level of TGF-β1 was significantly increased in endometrioma lesions compared to eutopic endometrial tissues from healthy controls which in consistent with previous study [40, 41] (Additional file 1: Fig. S3). To elucidate the functional role of KAT14 in endometrioma-associated fibrosis induced by TGF-β1, we constructed KAT14 overexpression immortalized human endometrial stromal cell line in which KAT14 expression level was assessed by qRT-PCR and western blotting assays. As shown in Additional file 1: Fig. S4, the infection of HESCs with a lentiviral vector containing *KAT14* cDNA (pCDH-*KAT14*) resulted in a notable increase in KAT14 mRNA and protein expression compared to the negative control (pCDH-Ctrl). To further analyze the functional consequences of KAT14 overexpression in HESCs, we performed RNA-seq on HESCs with KAT14 overexpression under TGF-β1 stimulation which due to the essential role of TGF-β1 within KAT14 mediated profibrotic response in HESCs according to our previous research (data not show). The heatmap and volcano plot showed 2456 up- and 2590 down-regulated genes (Adjusted *P-value* < 0.05, log2 [fold change] > 1) between *KAT14*-overexpressed HESCs and scrambled control all treated with TGF-β1 (Additional file 1: Fig. S5A, B, and Additional file 2: Table S6). Gene ontology (GO) analysis revealed that the upregulated genes in the KAT14 overexpression group were enriched in multiple pathways associated with the ECM structural constituents based on molecular function terms (Fig. 2A, Additional file 3: Table S7), and collagen components based on cellular component terms (Additional file 1: Fig. S5C, Additional file 4: Table S8). Similarly, gene set enrichment analysis (GSEA) showed a robust impact of KAT14 overexpression on genes involved in the ECM-associated (Fig. 2B) and collagen-related pathways (Additional file 1: Fig. S5D). Furthermore, various collagens and other ECM genes were among the significantly upregulated genes (Fig. 2C). These data suggest that the observed upregulation of KAT14 endometrioma tissues could promote fibroblast activation and ECM remodeling under TGF-β1 stimulation.

Building on these results, we delved deeper into the involvement of KAT14 in HESC activation and ECM component production under TGF-β1 stimulation. Our findings revealed a pronounced augmentation in the TGF-β1-induced expression of the myofibroblast activation marker, α-SMA, as well as ECM proteins such as fibronectin and collagen I, both at the transcriptomic and proteomic levels, when KAT14 was overexpressed in HESCs compared to negative control cells (pCDH-Ctrl HESCs) (Fig. 2D, E). Immunofluorescence staining corroborated these observations, with discernible increases in fibronectin, collagen I, and α-SMA expression in KAT14-overexpressing HESCs under TGF-β1 stimulation (Fig. 2F). Collectively, these results underscore the pivotal role of KAT14 in TGF-β1-mediated myofibroblast differentiation and ECM production. Furthermore, pCDH-*KAT14* HESCs showed a significantly heightened contractile capability in collagen gel matrix under TGF-β1 stimulation compared to pCDH-Ctrl HESCs (Fig. 2G). Subsequently, a wound healing assay was performed to assess the effect of KAT14 on the migration ability of HESCs with TGF-β1 treatment. As shown in Fig. 2H, KAT14 overexpression not only significantly enhanced HESC migration, but also facilitated expedited wound healing under TGF-β1 stimulation. In summary, the data demonstrate that KAT14 assumes a pivotal role in the activation, migration, and ECM synthesis of HESCs in vitro stimulated by TGF-β1.

### *KAT14* silencing alleviates profibrotic effects of TGF-β1 in primary human EcESCs and HESCs

To comprehensively interrogate the influence of KAT14 on TGF-β1-associated profibrotic effects on endometrioma lesions, we next systematically investigated primary human EcESCs. Authentication of these cells was ascertained through immunofluorescence assays, as depicted in Additional file 1: Fig. S6A. Two distinct and stable primary cell lines featuring *KAT14*-KD were established via the transfection of lentiviral vectors containing short hairpin RNA (shRNA)-*KAT14*, which were verified by qRT-PCR and western blotting (Additional file 1: Fig. S6B, C). As shown in Fig. 3, TGF-β1 instigated a time-dependent escalation in KAT14 expression along with the upregulation of profibrotic markers such as fibronectin, collagen I, and α-SMA (Fig. 3A). Notably, this effect was significantly mitigated in cells subjected to *KAT14*-KD when compared to the negative control (shRNA-Ctrl), irrespective of the presence or absence of TGF-β1 stimulation (Fig. 3B, C). Additionally, the results of immunofluorescence staining revealed a substantial inhibition of TGF-β1-induced upregulation of fibronectin, collagen I, and α-SMA in EcESCs subjected to *KAT14*-KD (Fig. 3D). Furthermore, functional assessments through collagen gel contraction and wound healing assays revealed that *KAT14* KD markedly attenuated the contractile capacity and migration ability potentiated by TGF-β1 on human EcESCs compared to negative control cells (Fig. 3E, F).

To further confirm the role of KAT14 in TGF-β1-mediated endometrioma-associated fibrogenesis, two independent *KAT14*-KO HESCs monoclonal cell lines were generated using the CRISPR-Cas9 technique, denoted as KAT14^#2^ and KAT14^#44^ HESCs (Additional file 1: Fig. S7). In line with our observations in *KAT14*-KD EcESCs, a significant reduction in the expression of fibronectin, collagen I and α-SMA were evident in *KAT14*-KO HESCs compared to wild-type cells with TGF-β1 treatment (Additional file 1: Fig. S8A–C). Moreover, the contractile and migratory capacities of *KAT14*-KO HESCs were significantly diminished compared to those of wild type cells under TGF-β1 stimulation (Additional file 1: Fig. S8D, E). Intriguingly, the restoration of KAT14 expression in *KAT14*-KO HESCs through transfection with the lentiviral vector pCDH-*KAT14* rescued the pro-fibrogenetic markers, collagen gel contraction capacity, and cell migration ability under TGF-β1 stimulation (Additional file 1: Fig. S9A–E), This rescue effect further substantiates the positive regulatory role of KAT14 in the profibrotic response of endometrial stromal cells under TGF-β1 stimulation. Taken together, these findings compellingly support the notion that KAT14 plays a pivotal role in the activation, migration, and ECM production of EcESCs induced by TGF-β1, thereby contributing to endometrioma-associated fibrosis.

### Silencing *KAT14* protects fibrogenesis of endometriosis in vivo

To ascertain the potential mediating role of KAT14 in endometriosis-associated fibrosis in an in vivo context, we established an endometriosis mouse model following previously described protocols [21]. Adeno-associated virus (AAV) carrying *KAT14*-shRNA (AAV9-sh*KAT14*) or control shRNA (AAV9-shCtrl) was used for targeted delivery to the ectopic tissue of endometriosis mice (Fig. 4A). Subsequent examination of KAT14 expression in ectopic lesions involved the use of qRT-PCR, western blotting, and IHC staining (Additional file 1: Fig. S10A–C). Following AAV9-shCtrl injection, ectopic lesions in endometriosis mice exhibited elevated levels of α-SMA, fibronectin, and collagen I at both mRNA and protein levels compared to the eutopic endometrium from AAV9-shCtrl-injected and sham-operated mice (Fig. 4B, C). Notably, injection with AAV9-sh*KAT14* attenuated the up-regulation of α-SMA, fibronectin, and collagen I in ectopic lesions from endometriosis mouse model (Fig. 4B, C). Furthermore, Masson’s trichrome staining and IHC showed that *KAT14* KD significantly prohibited collagen I deposition and α-SMA expression in ectopic lesions from endometriosis mice compared to those injected with AAV9-shCtrl (Fig. 4D). Immunofluorescence staining further showed a marked decrease in the expression of α-SMA, collagen I, and fibronectin in ectopic lesions from the *KAT14-KD* endometriosis mice (Fig. 4E). These findings collectively underscore the involvement of KAT14 in the regulation of endometriosis-associated fibrosis by decreasing myofibroblast activation and ECM synthesis in vivo.

### KAT14 mediates epigenetic activation of *ACTA2* in response to TGF-β1 in primary human EcESCs

**Fig. 5:**
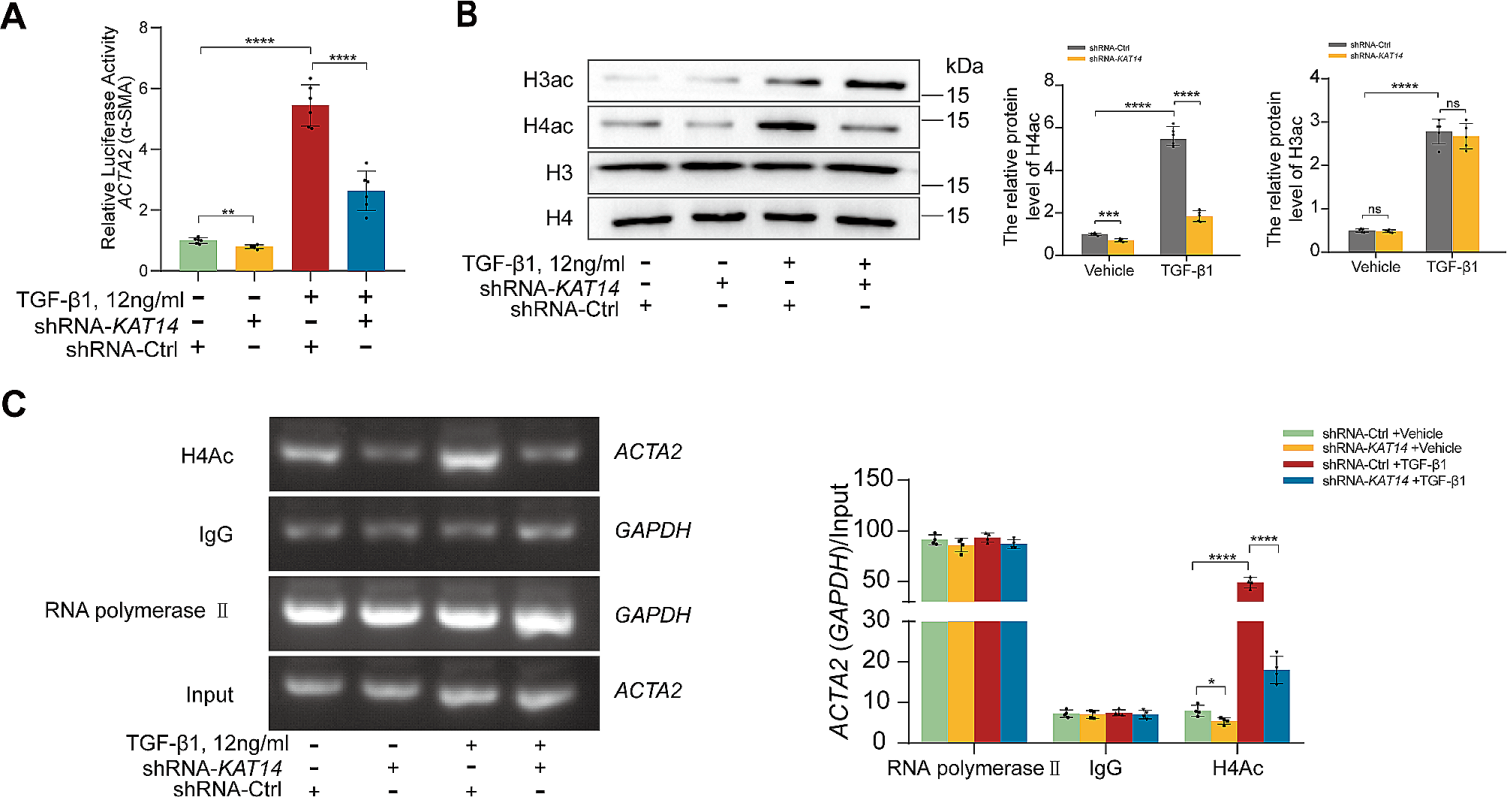
TGF-β1 induces KAT14-dependent *ACTA2* transcription in primary human EcESCs. (**A)** Luciferase reporter assay showing *ACTA2* promoter activity in primary EcESCs infected with shRNA-*KAT14* or shRNA-Ctrl lentiviruses and treated with TGF-β1 (12 ng/ml) or vehicle for 24 h. (**B)** Primary EcESCs (*n* = 6) infected with shRNA-*KAT14* or shRNA-Ctrl lentiviruses were treated with TGF-β1 or vehicle for 24 h. Western blot assay was performed to examine the effects of *KAT14* knockdown on total histone H3, histone H4, acetylated histone H3 (H3ac), and acetylated histone H4 (H4ac) levels. (**C)** ChIP-qPCR was used to analyze α-SMA promoter occupancy by H4ac in primary EcESCs (*n* = 4) infected with shRNA-*KAT14* or shRNA-Ctrl lentiviruses under treatment with TGF-β1 or vehicle for 24 h. The binding of RNA polymerase II and IgG to the *GAPDH* promoter was used as a positive and negative control, respectively. For stimulation, TGF-β1 was used at a concentration of 12 ng/ml. Data are representative of three or more independent experimental replicates. For all panels, data are presented as the mean ± SD. *P*-values were determined by Student’s t-test in panel (**B**), and one-way ANOVA in panels (**A**, **C**). *P < 0.05, **P < 0.01, ***P < 0.001, ****P < 0.0001, ns: Not significant, EcESCs: ectopic endometrial stromal cells, Ctrl: control, ChIP: chromatin immunoprecipitation

In our study, the luciferase reporter assay results showed a notable attenuation of TGF-β1-induced ACTA2 gene transcription in human EcESCs upon *KAT14* silencing, suggesting a pivotal role for KAT14 in TGF-β-induced α-SMA expression (Fig. 5A). However, the intricate mechanistic underpinnings of this regulatory phenomenon remain incompletely understood. KAT14 is a HAT that regulates target gene expression by mediating histone acetylation [42]. Our previous study showed that KAT14 promotes α-SMA expression by mediating the acetylation of histone H3 and histone H4 [18]. Therefore, we hypothesized that KAT14 facilitates *ACTA2* transcription in human endometrial stromal cells through histone modifications. To substantiate this hypothesis, we examined the histone acetylation levels within human EcESCs in the presence or absence of TGF-β1 for 24 h and found that TGF-β1 significantly increased the histone H3 acetylation (H3ac) and histone H4 acetylation (H4ac) levels, while *KAT14* silencing selectively impeded the increase in H4ac induced by TGF-β1 (Fig. 5B). These results imply that, under TGF-β stimulation, KAT14 predominantly influences H4ac levels in EcESCs. Subsequently, a ChIP assay was performed to further elucidate the role of KAT14 in TGF-β1-induced histone modification within the promoter regions of *ACTA2*. We found that TGF-β1 increased the H4ac levels in the promoter regions of *ACTA2*, while *KAT14*-KD significantly abrogated the upregulation of H4ac in the promoter regions of *ACTA2* induced by TGF-β1 (Fig. 5C). These results demonstrate the role of KAT14 in promoting α-SMA expression via acetylating H4ac under TGF-β stimulation in EcESCs.

### SRF was increased and bound to KAT14 in primary human EcESCs under TGF-β1 stimulation

**Fig. 6:**
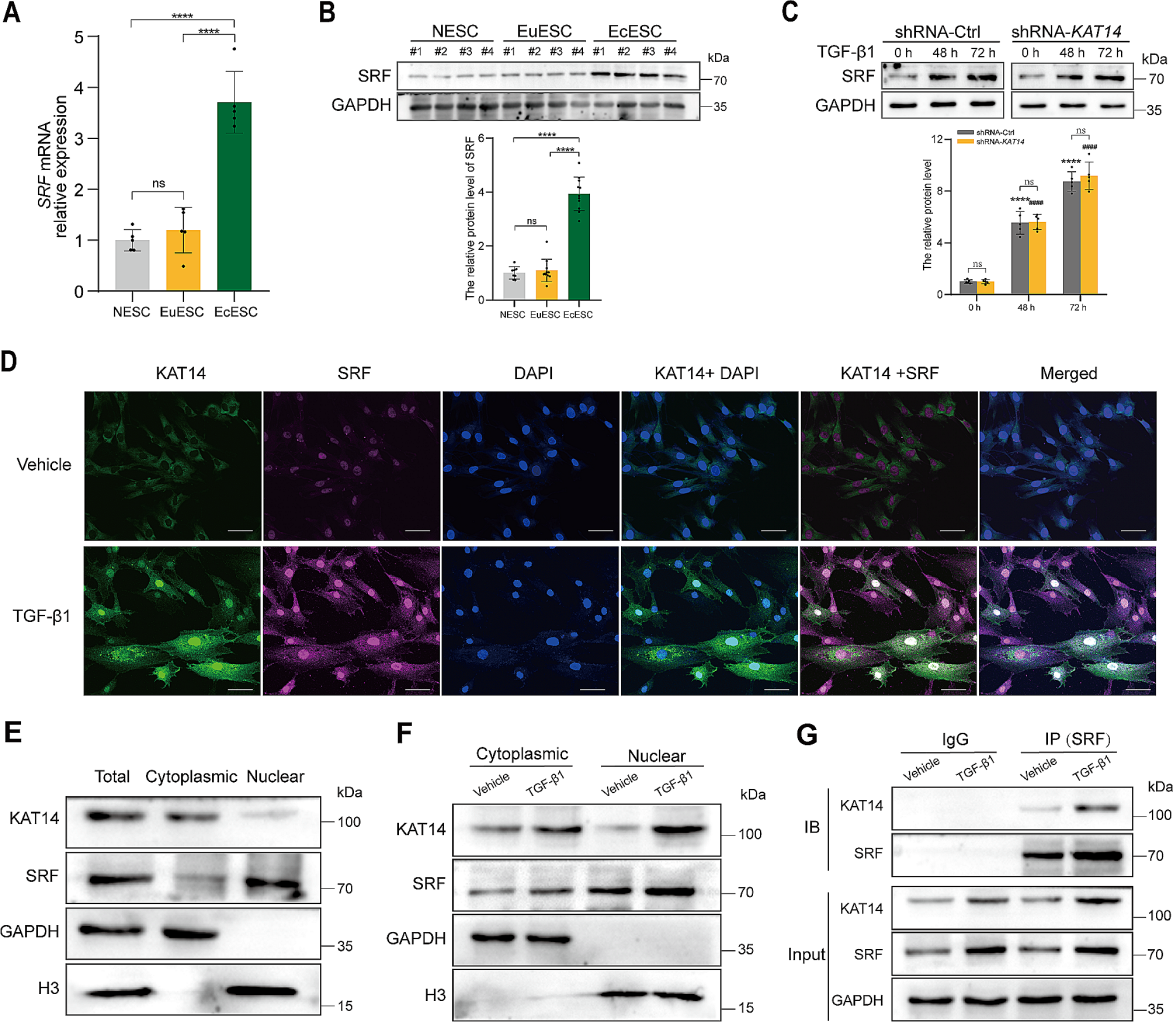
TGF-β1 increases SRF expression and promotes KAT14 nuclear translocation through co-operating with SRF in primary human EcESCs. (**A)** qRT-PCR analysis of *SRF* expression in primary NESCs (*n* = 5), EuESCs (*n* = 5), and EcESCs (*n* = 5). Relative quantification of gene expression was calculated using the 2^−∆∆Ct^ method and normalized to *GAPDH* as the internal control. (**B)** Western blotting assay analysis showing the SRF protein levels in primary NESCs (*n* = 8), EuESCs (*n* = 11), and EcESCs (*n* = 11). (**C)** Western blotting analysis showing the SRF protein level in primary human EcESCs infected with shRNA-*KAT14* or shRNA-Ctrl lentiviruses under TGF-β1 stimulation for 0, 48, and 72 h, respectively. ****P < 0.0001 versus shRNA-Ctrl at 0 h; ^####^*P* < 0.0001 versus shRNA-*KAT14* at 0 h. (**D)** Immunofluorescence staining assay showing the co-localization of KAT14 and SRF in primary EcESCs incubated with TGF-β1 or vehicle for 48 h. Scale bar: 50 μm. (**E)** Total cell lysate was prepared and cytoplasmic and nuclear proteins were isolated from primary EcESCs, and the proteins were subjected to immunoblotting with KAT14 and SRF antibodies. The purity of the cytoplasmic and nuclear fractions was confirmed by GAPDH and Histone-H3 antibodies. (**F)** Cytoplasmic and nuclear proteins were isolated from primary EcESCs treated with TGF-β1 or vehicle for 48 h, immunoblotting assay showing KAT14 and SRF expression in the cytosol and nucleus. (**G)** Immunoprecipitation with SRF antibodies shows pull down of KAT14 in primary EcESCs treated with or without TGF-β1, using isotype-matched immunoglobulin G (IgG) antibody as a negative control. For stimulation, TGF-β1 was used at a concentration of 12 ng/ml. Data are representative of three or more independent experimental replicates. For all panels, data are presented as the mean ± SD. *P*-values were determined by Student’s t-test in panel (**C**), and one-way ANOVA in panels (**A**–**C**). ****P < 0.0001, ns: Not significant, EcESCs: ectopic endometrial stromal cells, EuESCs: eutopic endometrial stromal cells, NESCs: normal endometrial stromal cells, Ctrl: control

To elucidate the precise mechanism by which KAT14 mediates TGF-β1-induced fibrogenesis, we hypothesized that SRF may serve as a crucial transcription factor for KAT14 in the modulation of TGF-β1-induced myofibroblast activation in human EcESC fibrogenesis. We found a significant increase in both mRNA and protein levels of SRF in EcESCs compared to human NESCs and EuESCs (Fig. 6A, B). Moreover, we demonstrated that SRF expression was markedly upregulated under TGF-β1 stimulation, and notably, this upregulation remained unaffected by *KAT14*-KD in EcESCs (Fig. 6C).

We next performed an immunofluorescence assay to assess the expression and subcellular distribution of KAT14 and SRF in human EcESCs subjected to TGF-β1 stimulation. The results showed a significant augmentation in nuclear SRF and KAT14 protein levels under TGF-β1 stimulation (Fig. 6D). Subsequently, to further elucidate the role and precise mechanism underlying the KAT14-mediated TGF-β1-induced fibrogenesis in EcESCs with SRF, we examined the expression of nuclear and cytosolic SRF and KAT14 (Fig. 6E). Western blotting analysis demonstrated a substantial increase in nuclear SRF and KAT14 protein levels in response to TGF-β1 stimulation (Fig. 6F), suggesting that TGF-β1 stimulation promotes KAT14 and SRF nuclear translocation. Consistent with our earlier immunofluorescence assay, co-localization of KAT14 with SRF was observed in the nucleus. We further revealed the physical interactions of endogenous SRF with KAT14 by performing a co-immunoprecipitation assay (Fig. 6G). We confirmed the presence of the KAT14/SRF complex by immunoprecipitation following nuclear-cytoplasmic separation of TGF-β1-stimulated EcESCs (Additional file 1: Fig. S11). Collectively, these results indicate that SRF significantly increased in response to TGF-β1, interacted with KAT14, and subsequently translocated into the nucleus in primary human EcESCs under TGF-β1 stimulation. These findings suggest a pivotal role for SRF in the KAT14-mediated TGF-β-associated fibrotic signaling pathway in human EcESCs.

### KAT14/SRF transcription activator complex is essential for TGF-β1-induced fibrogenesis in primary human EcESCs

**Fig. 7:**
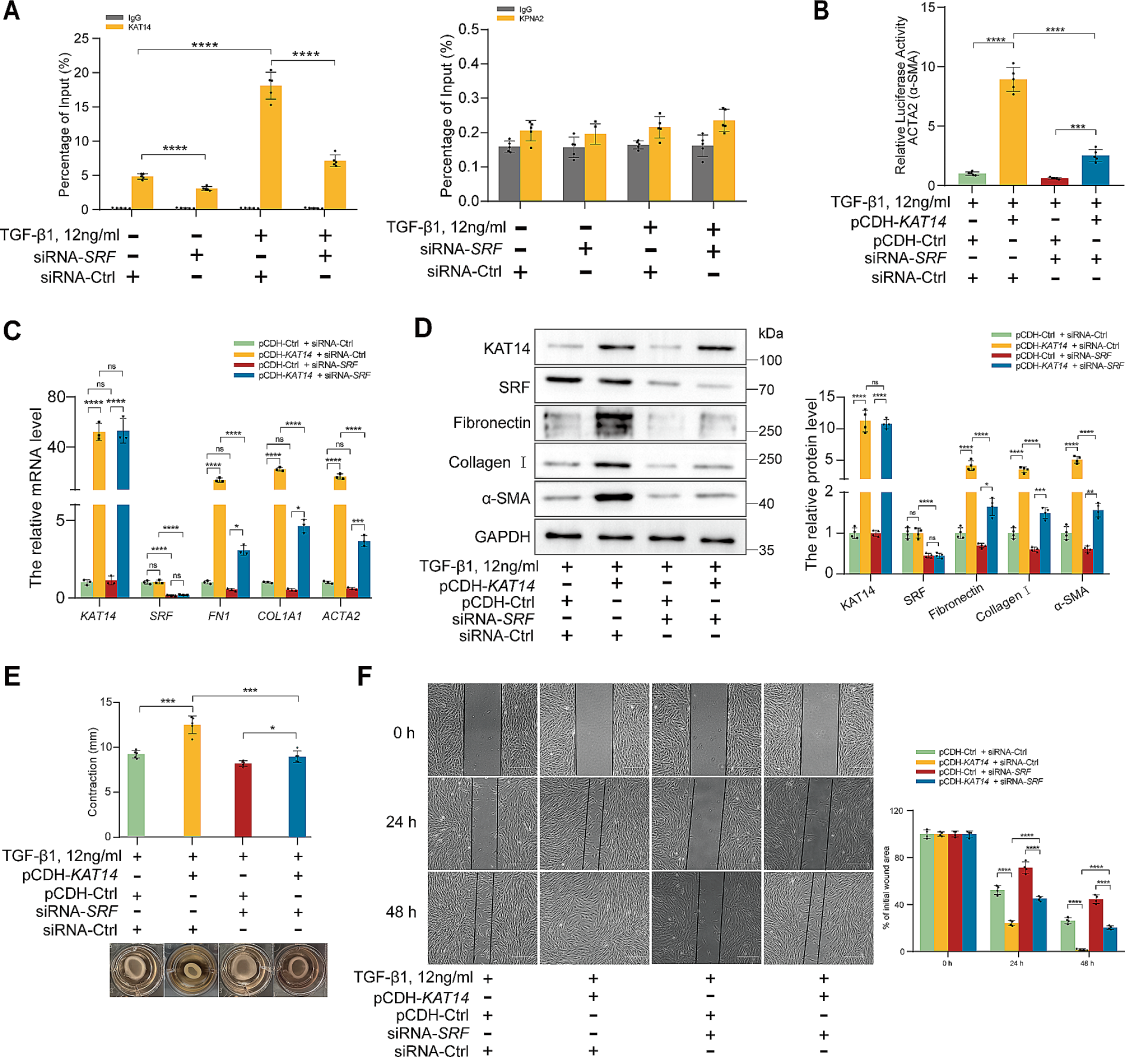
KAT14-mediated TGF-β1-induced fibrogenic responses are regulated through SRF. (**A)** ChIP assay showing the binding of KAT14 to the CArG box of the *ACTA2* promoter (left panel) in primary human EcESCs co-transfected with siRNA-*SRF* or siRNA-Ctrl under stimulation with TGF-β1 or vehicle for 24 h. The *KPNA2* promoter (right panel) served as the negative control. (**B)***KAT14*-KD primary EcESCs infected with pCDH-*KAT14* or pCDH-Ctrl lentiviruses in the presence of siRNA-*SRF* or siRNA-Ctrl stimulated with TGF-β1 and then subjected to luciferase reporter assay. (**C)** qRT-PCR analysis showing the mRNA levels of *FN1*, *COL1A1*, *ACTA2, KAT14*, and *SRF* in *KAT14*-KD primary EcESCs infected with pCDH-*KAT14* or pCDH-Ctrl lentiviruses in the presence of siRNA-*SRF* or siRNA-Ctrl stimulated with TGF-β1 for 24 h. Relative quantification of gene expression was calculated using the 2^−∆∆Ct^ method and normalized to *GAPDH* as the internal control. (**D)** Immunoblotting analysis showing the protein levels of fibronectin, collagen 1, KAT14, SRF, and α-SMA in *KAT14*-KD primary EcESCs infected with lentiviruses harboring pCDH-*KAT14* or pCDH-Ctrl vectors in the presence of siRNA-*SRF* or siRNA-Ctrl stimulated with TGF-β1 for 24 h. (**E**, **F)** Collagen gel contractility and wound healing assays were performed to assess (**E**) the cell contraction capacity and (**F**) cell migration ability of *KAT14*-KD primary EcESCs infected with pCDH-*KAT14* or pCDH-Ctrl lentiviruses in the presence of siRNA-*SRF* or siRNA-Ctrl stimulated with TGF-β1 (collagen gel contractility assay for 24 h; wound healing assay for 0, 24, and 48 h). Scale bar: 200 μm. The concentration of TGF-β1 used for stimulation was 12 ng/ml. Data are representative of three or more independent experimental replicates. For all panels, data are presented as the mean ± SD. *P*-values were determined by one-way ANOVA test. *P < 0.05, **P < 0.01, ***P < 0.001, ****P < 0.0001, ns: Not significant, Ac: acetylation, EcESCs: ectopic endometrial stromal cells, Ctrl: control, ChIP: chromatin immunoprecipitation, KD: knockdown

To further elucidate the involvement of SRF in KAT14-mediated TGF-β-induced α-SMA expression in primary human EcESCs, ChIP was conducted using an anti-KAT14 antibody, followed by qRT-PCR for bound DNA. The results revealed that KAT14 was recruited to the *ACTA2* promoter sequence [CC(A/T)_6_GG] (CArG box, also known as serum response element), to which SRF binds, thereby initiating the expression of fibrosis-associated genes under TGF-β1 stimulation [19, 30] (Fig. 7A). To investigate the role of SRF in mediating TGF-β1-induced myofibroblast activation by KAT14, we next employed SRF siRNA to knockdown SRF expression in EcESCs. As a result, *SRF* KD significantly prohibited the binding of KAT14 to the CArG box of the *ACTA2* promoter in EcESCs (Fig. 7A). Furthermore, the luciferase reporter assay results showed that the counteractive effect of KAT14 re-enhancement on TGF-β1-induced *ACTA2* transcription in *KAT14*-KD EcESCs was attenuated by *SRF* KD (Fig. 7B). Subsequently, qRT-PCR was performed to explore the role of SRF in KAT14-mediated TGF-β1-induced myofibroblast activation and ECM synthesis in human EcESCs. The results showed that the TGF-β1-induced increase in α-SMA, collagen I, and fibronectin expression, mediated by re-overexpression of *KAT14* in *KAT14*-KD EcESCs, was significantly abrogated by *SRF*-KD (Fig. 7C), a finding further confirmed by western blotting (Fig. 7D). Moreover, *SRF*-KD impedes TGF-β1-induced collagen gel contractility and migration mediated by the re-enhancement of KAT14 in *KAT14*-KD human EcESCs (Fig. 7E, F).

To further confirm the importance of SRF in KAT14-mediated fibrogenesis, we used CCG-1423, a specific SRF pathway inhibitor. Consistent with our previous results, SRF inhibition significantly attenuated the expression of pro-fibrotic markers resulting from KAT14 re-enhancement in *KAT14*-KD EcESCs under TGF-β1 stimulation (Additional file 1: Fig. S12). Collectively, these findings demonstrate the essential role of SRF in KAT14-mediating profibrotic effects. The KAT14/SRF complex serves as a key regulator in endometrioma-associated fibrogenesis by mediating the TGF-β-induced EcESC activation and ECM protein synthesis, suggesting that SRF may be a therapeutic target of KAT14-mediated endometrioma-associated fibrogenesis.

### KAT14/SRF complex is associated with increased α-SMA expression in ovarian endometrioma lesions

**Fig. 8:**
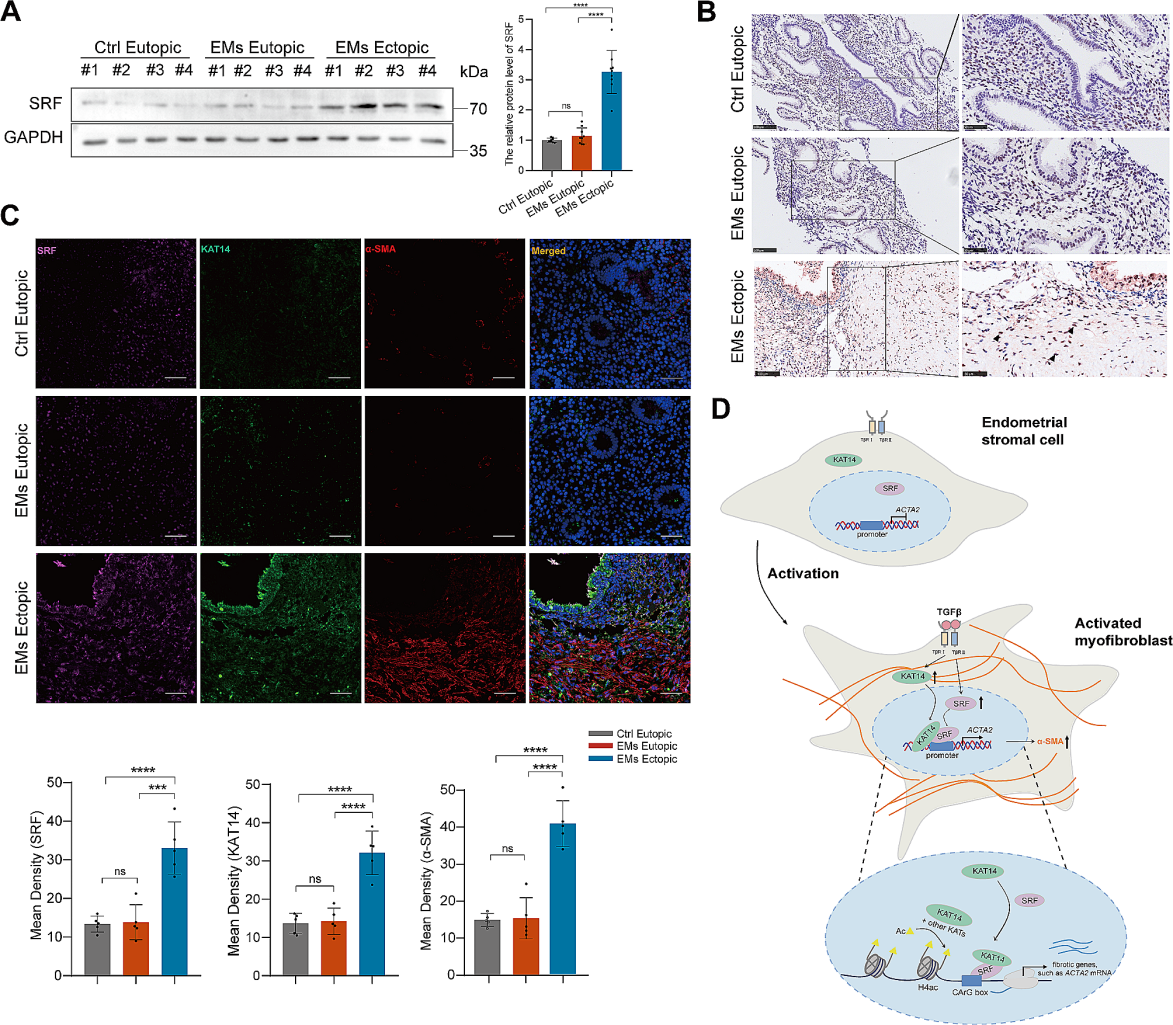
Aberrant expression of SRF co-localizing with KAT14 was found in ovarian endometrioma lesions. (**A)** Western blot analysis of SRF protein expression in the eutopic endometrial tissues from healthy controls (*n* = 8), ectopic lesions (*n* = 9), and eutopic endometrial (*n* = 9) tissues from patients with endometrioma. (**B)** Representative images of SRF immunohistochemistry staining in the eutopic endometrial tissues from healthy controls (*n* = 8), ectopic lesions (*n* = 9), and eutopic endometrial (*n* = 9) tissues from patients with endometrioma. Bar: 100 μm (left panel), 50 μm (right panel); arrows indicate positive SRF expression. (**C)** Representative triple immunofluorescence staining images of α-SMA, SRF, and KAT14 protein in the eutopic endometrial tissues from healthy controls (*n* = 5), ectopic lesions (*n* = 5), and eutopic endometrial (*n* = 5) tissues from patients with endometrioma. Bar: 50 μm. (**D)** Schematic summary of the proposed profibrotic mechanisms by which KAT14 contributes to the pathogenesis of endometrioma-associated fibrosis. TGF-β1 stimulation of primary human EcESCs increases KAT14 and SRF expression, induces the interaction between KAT14 and SRF, and the acetylation of histone H4 at *ACTA2* promoter regions to activate α-SMA expression, thus promoting EcESC transition into myofibroblasts, along with ECM deposition, ultimately resulting in endometrioma-associated fibrosis. Data are representative of three or more independent experimental replicates. For all panels, data are presented as the mean ± SD. *P*-values were determined through one-way ANOVA. ***P < 0.001, ****P < 0.0001, ns: Not significant, Ac: acetylation, Ctrl: control, EcESCs: ectopic endometrial stromal cells, EMs: endometriomas

To gain further insights into the involvement of the KAT14/SRF complex in ovarian endometrioma-associated fibrosis, we next assessed its levels in ectopic lesions of patients with ovarian endometrioma. The results of western blotting assay demonstrated a significant elevation in SRF protein levels within ectopic lesions compared to the eutopic endometrium from patients with ovarian endometrioma and healthy controls (Fig. 8A). Furthermore, IHC studies showed a pronounced increase in SRF intensity in sections from ovarian endometrioma ectopic lesions relative to eutopic endometrium sections from patients with ovarian endometrioma and healthy controls (Fig. 8B). Moreover, immunofluorescent staining revealed a notable augmentation in SRF expression within ovarian endometrioma lesions, primarily co-localizing with KAT14 in α-SMA-positive myofibroblast cells (Fig. 8C). Taken together, these results indicate a significant upregulation of SRF in ectopic lesions of ovarian endometrioma, with predominant co-localization with KAT14 in myofibroblasts, underscoring the pivotal role of the KAT14/SRF complex in TGF-β-induced myofibroblast activation during the fibrogenesis of ovarian endometrioma (Fig. 8D).

## Discussion

Although significant progress has been made in understanding the precise mechanisms underlying TGF-β-induced endometriosis-associated fibrosis [43, 44], there remains a critical need to develop therapeutic strategies with high efficiency and low adverse effects for the treatment of TGF-β-associated fibrosis in ectopic lesions of endometriosis, especially for endometriomas. This study elucidates the pivotal role of KAT14, an important HAT protein, in TGF-β-induced fibrosis within endometrioma lesions. The elevated expression of KAT14 in ectopic lesions from patients with endometrioma contributes to fibrogenesis in primary human EcESCs through enhancing the profibrotic effects of TGF-β on over-activation of myofibroblasts and excessive production of ECM proteins. Mechanically, KAT14 promotes α-SMA expression through histone tail modification at *ACTA2* promoter regions via forming a complex with SRF, which is significantly upregulated in ectopic lesions in endometrioma under TGF-β1 stimulation. Additionally, siRNA knockdown or the pharmacological inhibition of SRF was found to attenuate KAT14-mediated myofibroblast activation and pro-fibrotic responses induced by TGF-β1 in vitro. Moreover, *KAT14* knockdown in an endometriosis mouse model using adeno-associated viruses significantly prohibited endometriosis-associated fibrosis in vivo. These findings reveal a novel pathogenetic role of KAT14 in the fibrogenesis of endometriosis through TGF-β-associated fibrosis signaling via cooperation with SRF. Targeting the KAT14/SRF complex emerges as a potential therapeutic approach for endometriosis-associated fibrosis, especially in endometriomas.

TGF-β functions as a key cytokine in the initiation and development of fibrotic diseases [45, 46], triggering fibrosis through SMAD2/3-dependent canonical TGF-β signaling or SMAD-independent noncanonical TGF-β signaling [47, 48]. Previous studies have revealed a significant increase in TGF-β1 expression in ectopic lesions in endometriosis and in peritoneal fluids from patients with endometriosis [49–51]. Numerous studies have shown that TGF-β contributes to the pathogenetic process of fibrosis in endometriosis by inducing fibroblast-to-myofibroblast transition, as well as myofibroblast proliferation and overactivation, thus promoting ECM protein deposition in ectopic lesions [37, 52]. Despite the potential of TGF-β signaling inhibition as a strategy for fibrosis diseases, the direct targeting of TGF-β pathways has failed due to the essential physiological role of TGF-β signaling [53–55]. Indeed, inhibiting TGF-β signaling could lead to adverse reactions and impaired physical functions [56, 57]. Thus, revealing the precise mechanisms responsible for mediating TGF-β-induced profibrotic effects is crucial for developing efficient and low-adverse-effect therapeutic targets for fibrosis diseases, such as endometriosis.

Histone acetylation, an epigenetic modification promoted by HATs, plays a key role in the transcriptional regulation of α-SMA and ECM molecules by altering the conformation of heterochromatin [58–60]. Recent studies have demonstrated that the dysregulated expression of HATs promotes TGF-β-induced fibrosis [14]. Selective inhibition of the acetyltransferase activity of HAT with a small molecule significantly reduces the TGF-β profibrotic effects on fibroblasts, suggesting that HATs are a potential therapeutic target for the TGF-β signaling pathway [61–63]. As we previously reported, KAT14 is one of the HATs that plays a key role in mediating *ACTA2* expression in smooth muscle cells [18]. However, the role of KAT14 in TGF-β-induced fibrosis in endometriosis, especially ovarian endometrioma, remains unknown. We reveal elevated KAT14 expression in ectopic lesions from patients with ovarian endometrioma, predominantly in activated fibroblasts according to scRNA-seq analysis. Moreover, we demonstrated the presence of TGF-β-induced upregulation of KAT14 in immortalized HESCs and primary human EcESCs. Functional experiments and RNA-seq of HESCs with KAT14 overexpression confirm its crucial role in promoting TGF-β1-induced myofibroblast activation and profibrotic effects. Knockdown of KAT14 in primary human EcESCs attenuates TGF-β-induced profibrotic effects, further confirmed by CRISPR/Cas9-mediated KAT14 knockout in HESCs. AAV9-sh*KAT14* injection into an endometriosis mouse model significantly diminishes endometriosis-associated fibrogenesis. Collectively, the results of our in vitro and in vivo studies establish KAT14 as a key mediator in TGF-β-induced endometriosis-associated fibrosis.

The overactivation of myofibroblasts, characterized by α-SMA upregulation, is the main pathophysiological feature of fibrogenesis in ovarian endometrioma [52, 64]. We found that KAT14 is necessary for transcriptional activity and protein expression of α-SMA, suggesting its key regulatory role in TGF-β1-induced α-SMA expression. Previous work demonstrated that KAT14 promotes α-SMA expression by enhancing histone H3 and H4 hyperacetylation in smooth muscle cells [18]. However, whether KAT14 mediates ACTA2 gene expression in primary human EcESCs through histone acetylation was not revealed. Our findings reveal that TGF-β1 elevated the level of H4 acetylation on the *ACTA2* promoter in primary human EcESCs, the effects of which were abrogated by *KAT14*-knockdown. Moreover, the ChIP-seq results shows that TGF-β significantly promotes KAT14 binding to *ACTA2* promoter regions. These results suggest that KAT14 mediates α-SMA expression through binding to *ACTA2* promoter regions and promoting H4 acetylation.

Previous studies, including our own, have consistently demonstrated that KAT14 plays a pivotal role in mediating target gene transcription through translocating into the nucleus and binding to the promoter or enhancer regions of target genes. This occurs through cooperation with various co-activators, such as transcriptional factors [18, 65]. Our previous study also unveiled that KAT14 forms a complex with SRF, GATA6, and CRP to regulate the expression of smooth muscle genes [18]. SRF is a highly conserved transcription factor, which has a critical role in regulating the expression of smooth muscle genes [18, 66, 67]. It has also been reported that SRF is a key transcription factor contributing to the development of fibrotic diseases [19, 68]. However, whether KAT14-mediates TGF-β1-induced α-SMA expression in primary human EcESCs through interaction with SRF remains unknown. To elucidate the precise mechanism underlying the KAT14-mediated regulation of TGF-β1-induced fibrogenesis in ovarian endometrioma, we investigated the expression pattern of the KAT14/SRF axis. We found a significant increase in SRF expression in primary human EcESCs compared to NESCs and EuESCs. TGF-β1 promotes both SRF transcription and protein levels independently of KAT14 expression level. A series of experiments consistently demonstrated that KAT14 directly interacts with SRF in both cytosol and the nucleus. Moreover, TGF-β1 promotes KAT14/SRF complex translocation to the nucleus, where it binds to the promoter regions of *ACTA2*, triggering α-SMA expression. This process leads to the differentiation of fibroblasts into myofibroblasts and the subsequent excessive production of ECM molecules in primary human EcESCs under TGF-β stimulation (Fig. 8D). Moreover, knocking down SRF expression or pharmacologically inhibiting SRF significantly abrogates the profibrotic responses mediated by KAT14 overexpression in primary human EcESCs under TGF-β1 stimulation. This further supports the idea that the KAT14/SRF complex plays an essential role in TGF-β1*-*induced fibrogenesis in endometrioma. Collectively, these results underscore the significance of SRF as the key co-activator for KAT14, and the formation of the KAT14/SRF axis emerges as the underlying mechanism by which KAT14 mediates TGF-β1-induced fibrosis in endometriomas. This axis represents a potential therapeutic target for addressing the progression of endometrioma-associated fibrogenesis.

Finally, to confirm the pathogenetic importance of the KAT14/SRF axis in endometrioma, we conducted a thorough analysis of the expression pattern of the KAT14/SRF complex in patients with endometrioma. Our findings revealed a substantial increase in the expression level of the KAT14/SRF complex within ectopic lesions from patients with endometrioma compared to eutopic endometrial specimens from healthy controls and from patients with endometrioma. Importantly, this elevation was positively correlated with over-expression of α-SMA.

Nonetheless, our study is subject to several limitations. First, the in vitro investigation into the profibrotic role of KAT14 involved constructing KAT14 knockout immortalized HESCs through CRISPR-Cas9, as well as KAT14 knockdown in primary human EcESCs via transfection with shRNA-*KAT14* lentiviruses. While KAT14 knockout in primary human EcESCs could offer more compelling evidence, the intricate process of constructing *KAT14*-KO cells through CRISPR-Cas9 spanning 14–21 days may lead to alterations in the original characteristics of primary human EcESCs. Second, while we evaluated the role of KAT14 in endometriosis-associated fibrosis in vivo by transfecting ectopic endometrial tissues with AAV9-sh*KAT14*, a more robust approach would involve the use of endometrial stromal cell Kat14-specific knockout mice.

## Conclusion

In conclusion, our results demonstrate that TGF-β1-induced KAT14 in EcESCs plays a pivotal role in mediating TGF-β-associated profibrotic effects. This is achieved through the promotion of α-SMA and ECM protein synthesis facilitated by the interaction with SRF. The inhibition of KAT14 and its co-activators, such as SRF, has emerged as a promising avenue for therapeutic intervention in the treatment of endometriosis-associated fibrosis.

### Electronic supplementary material

Below is the link to the electronic supplementary material.

Supplementary Material 1

Supplementary Material 2

Supplementary Material 3

Supplementary Material 4

## Data Availability

All data have been shown in the main manuscript and supplemental materials. Data supporting the findings are available from the corresponding author upon reasonable request.

## Abbreviations

AAV: Adeno-associated virus
ATAC: Ada2a-containing
α-SMA: Alphasmooth muscle actin
ChIP: Chromatin immunoprecipitation
Co-IP: Coimmunoprecipitation
EcESCs: Ectopic endometrial stromal cells
ECM: Excessive extracellular matrix
ER: Estrogen receptor
ESCs: Endometrial stromal cells
EuESCs: Eutopic endometrial stromal cells
Go: Gene ontology
GSEA: Gene set enrichment analysis
H3ac: Histone H3 acetylation
H4ac: Histone H4 acetylation
HATs: Histone acetyltransferases
HDACs: Histone deacetylases
HESC: Human endometrial stromal cell
IHC: Immunohistochemistry
KAT14: Lysine acetyltransferase 14
KD: Knockdown
KO: Knockout
OE: Ovarian endometrioma
PBS: Phosphate buffered saline
PR: Progesterone receptor
qRT-PCR: quantitative reverse transcription PCR
RNA-seq: RNA sequencing
scRNA-seq: single-cell transcriptomes
sgRNA: single guide RNA
shRNA: short hairpin RNA
siRNA: small interfering RNA
SRF: Serum response factor
TGF-β: Transforming growth factor-β
UMIs: Unique molecular identifiers
