# Supplementary material for: Lysine acetyltransferase 14 mediates TGF-β-induced fibrosis in ovarian endometrioma via co-operation with serum response factor
Source: J Transl Med. 2024 Jun 12;22:561. doi: 10.1186/s12967-024-05243-2 (PMC11167823; doi:10.1186/s12967-024-05243-2)
Supplement: Supplementary file 1 — Supplementary Material 1 [file 12967_2024_5243_MOESM1_ESM.docx]

**Additional file 1**

**Additional Figures**


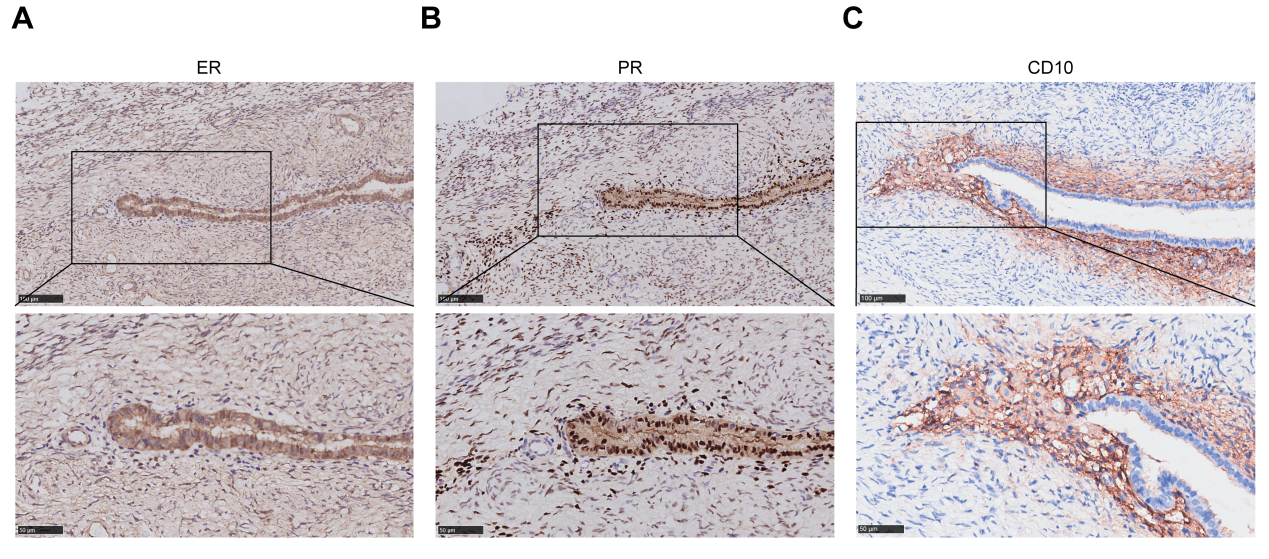


**Figure S1. Immunohistochemical staining of ER, PR and CD10 in ovarian endometrioma.** Representative immunohistochemical staining for ER (**A**), PR (**B**) and CD10 (**C**) in ovarian endometrioma. Scale bar: 100 μm (upper panel), scale bar: 50 μm (lower panel). ER: estrogen receptor; PR: progesterone receptor.


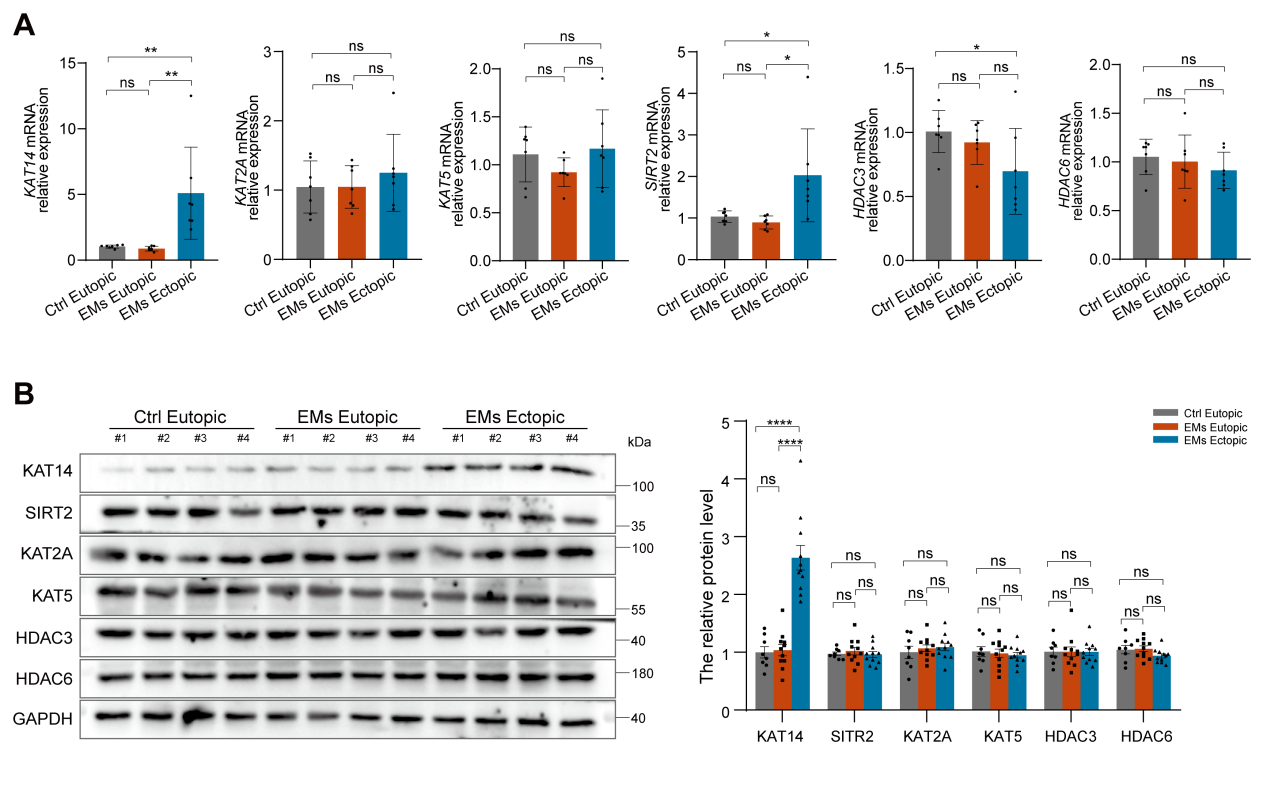


**Figure S2.** **Increased expression of KAT14 in ovarian endometrioma lesions.** (**A**) qRT-PCR analysis of the relative mRNA expression of *KAT14*, *SIRT2, KAT2A, KAT5, HDAC3*, and *HDAC6* in eutopic endometrial tissues from healthy controls (n = 7), ectopic lesions (n = 7), and eutopic endometrial (n = 7) tissues from patients with endometrioma. Relative quantification of gene expression was calculated using the 2^−△△Ct^ method and normalized to *GAPDH* as the internal control. (**B**) Western blot measurement of the protein level of KAT14, SIRT2, KAT2A, KAT5, HDAC3, and HDAC6 in eutopic endometrial tissues from healthy controls (n = 8), ectopic lesions (n = 9), and eutopic endometrial (n = 9) tissues from patients with endometrioma. Data are representative of three or more independent experimental replicates. Data are presented as the mean ± SD. *P*-values were determined by one-way ANOVA. **P* < 0.05, ***P* < 0.01, *****P* < 0.0001, ns: Not significant, EMs: endometriomas.


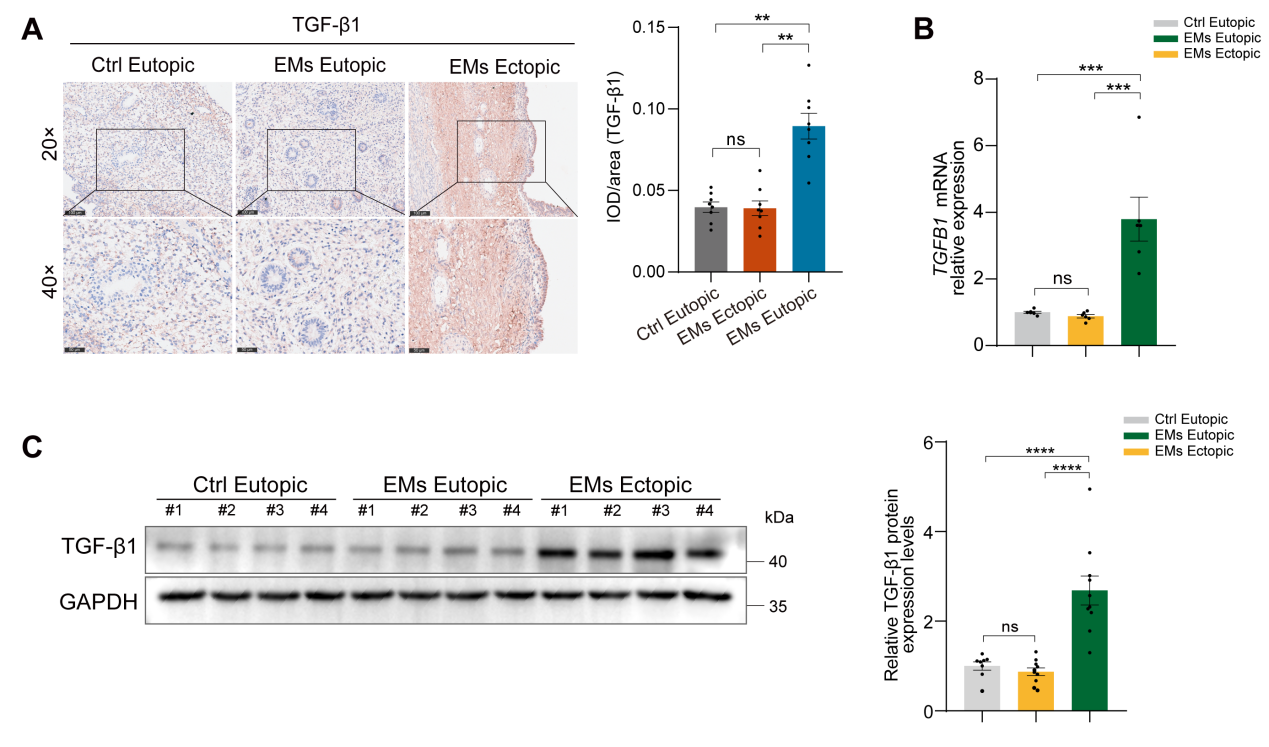


**Figure S3. TGF-β1 is increased in ovarian-endometrioma lesions.** (**A**) Representative immunohistochemical staining for TGF-β1 in human normal endometrium tissue (n = 8), eutopic endometrium (n = 8), and ectopic lesions (n = 8) from human ovarian endometrioma. Scale bar: 100 μm (upper panel), scale bar: 50 μm (lower panel). (**B**) qRT-PCR analysis of the relative mRNA expression of *TGFB1* in the eutopic endometrial tissues from healthy controls (n = 6), ectopic lesions (n = 6), and eutopic endometrial (n = 6) tissues from patients with endometrioma. Relative quantification of gene expression was calculated using the 2−△△Ct method and normalized to *GAPDH* as the internal control. (**C**) Western blot analysis of TGF-β1 protein expression in the eutopic endometrial tissues from healthy controls (n = 10), ectopic lesions (n = 10), and eutopic endometrial (n = 10) tissues from patients with endometrioma. Data are representative of three or more independent experimental replicates. Data are presented as the mean ± SD. *P*-values were determined by one-way ANOVA. ***P* < 0.01, ****P* < 0.001, *****P* < 0.0001, ns: Not significant, Ctrl: control, EMs: endometriomas.


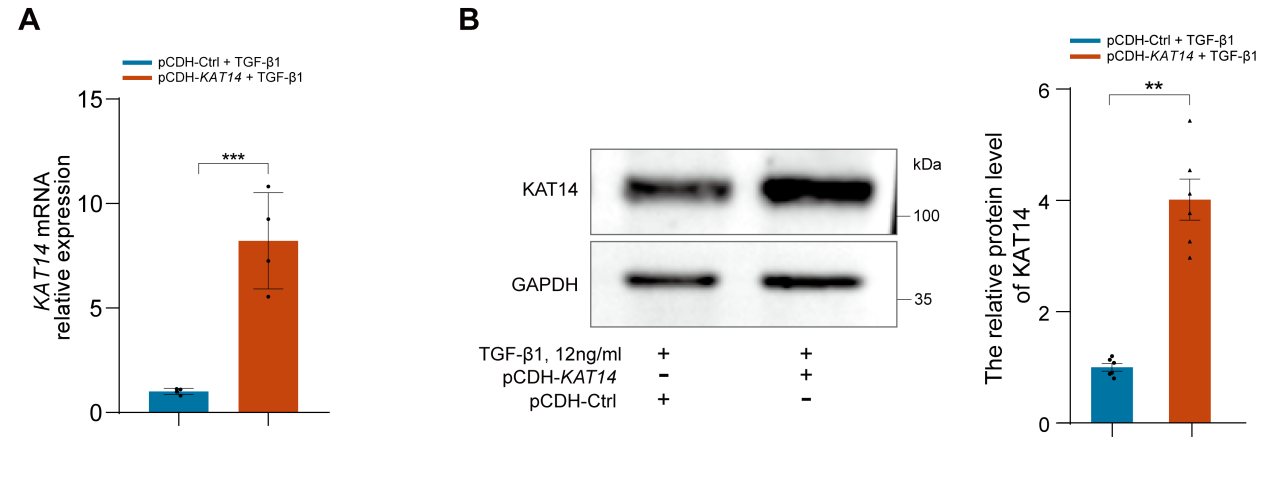


**Figure S4. pCDH-*KAT14* significantly increased KAT14 expression in HESCs.** (**A**) qRT-PCR analysis of the relative mRNA expression of *KAT14* in HESCs infected with the indicated lentiviruses harboring *KAT14* expression vector (pCDH-*KAT14*) (n = 4) or empty vector control (pCDH-Ctrl) (n = 4) treated with TGF-β1. Relative quantification of gene expression was calculated using the 2^−△△Ct^ method and normalized to *GAPDH* as the internal control. (**B**) Western blots measuring the protein level of KAT14 in HESCs infected with pCDH-*KAT14* or pCDH-Ctrl lentiviruses stimulated by TGF-β1. Data are representative of three or more independent experimental replicates. For all panels, data are presented as the mean ± SD. *P*-values were determined by Student’s t-test. ***P* < 0.01 and ****P* < 0.001.


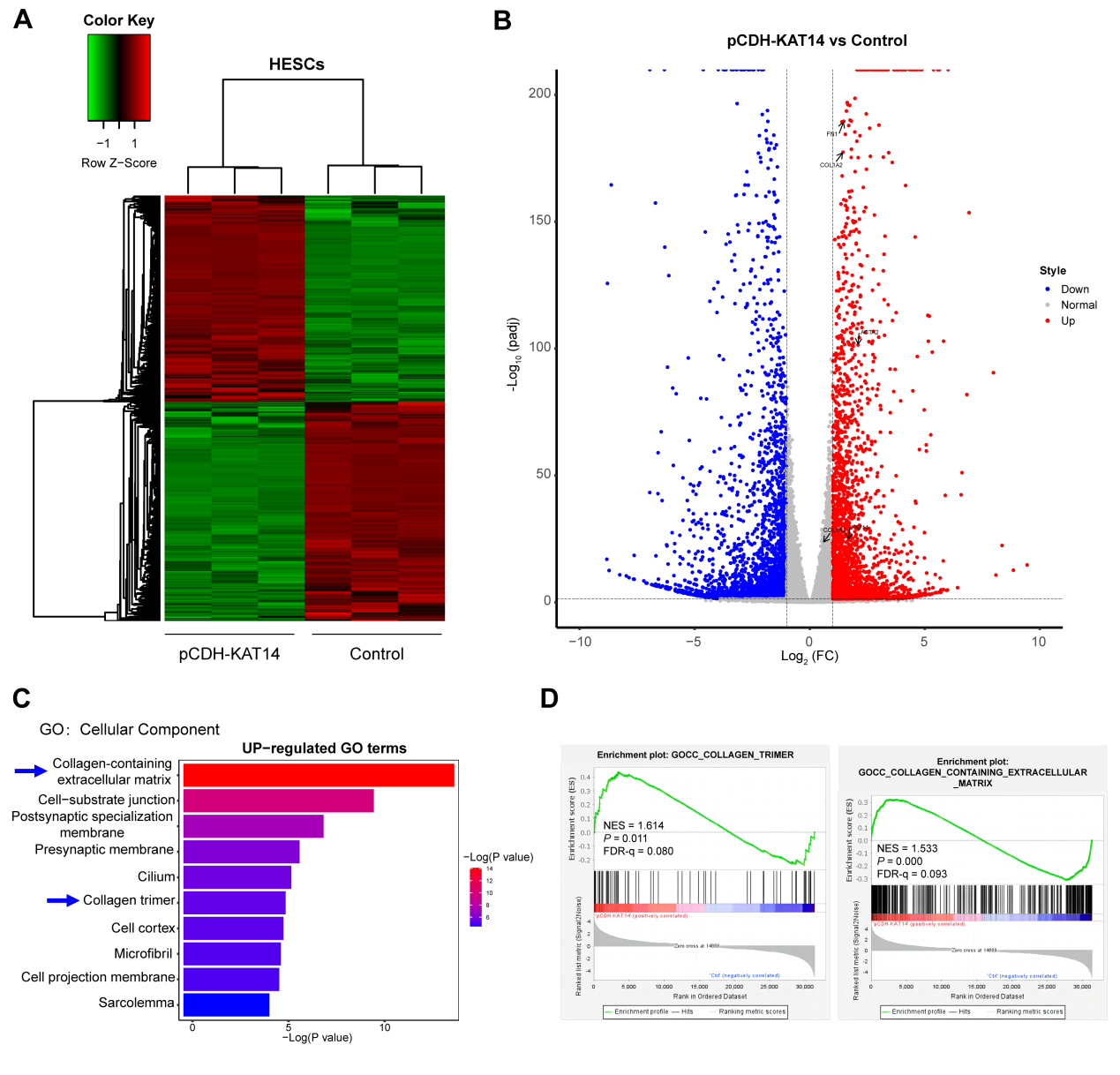


**Figure S5. KAT14 is involved in TGF-β1-induced fibrogenesis.** (**A**) Heatmap profile showing differentially expressed genes (DEGs) in HESCs infected with lentiviral vector containing *KAT14* cDNA (pCDH-*KAT14*) or negative control (pCDH-Ctrl) (n = 3 samples per group). (**B**) Volcano plot of DEGs in HESCs infected with pCDH-*KAT14* or pCDH-Ctrl lentiviruses reveals the myofibroblast activation marker among the genes most increased with KAT14 overexpression. Thresholds are shown as dashed gray lines; genes (dots) with significant differential expression are shown in red (up) or blue (down). Adjusted *P-value* < 0.05, log2 [fold change] > 1. (**C**) Bar graph showing the enrichment analysis of upregulated genes after KAT14 overexpression in terms of GO biological process. (**D**) Gene set enrichment analysis (GSEA) of DEGs induced by KAT14 overexpression compared to negative control (pCDH-Ctrl) shows significant enrichment in gene sets associated with collagen-related pathways (collagen trimer, collagen containing extracellular matrix). Normalized enrichment score (NES), false discovery rate (FDR), and *P-*values are shown.


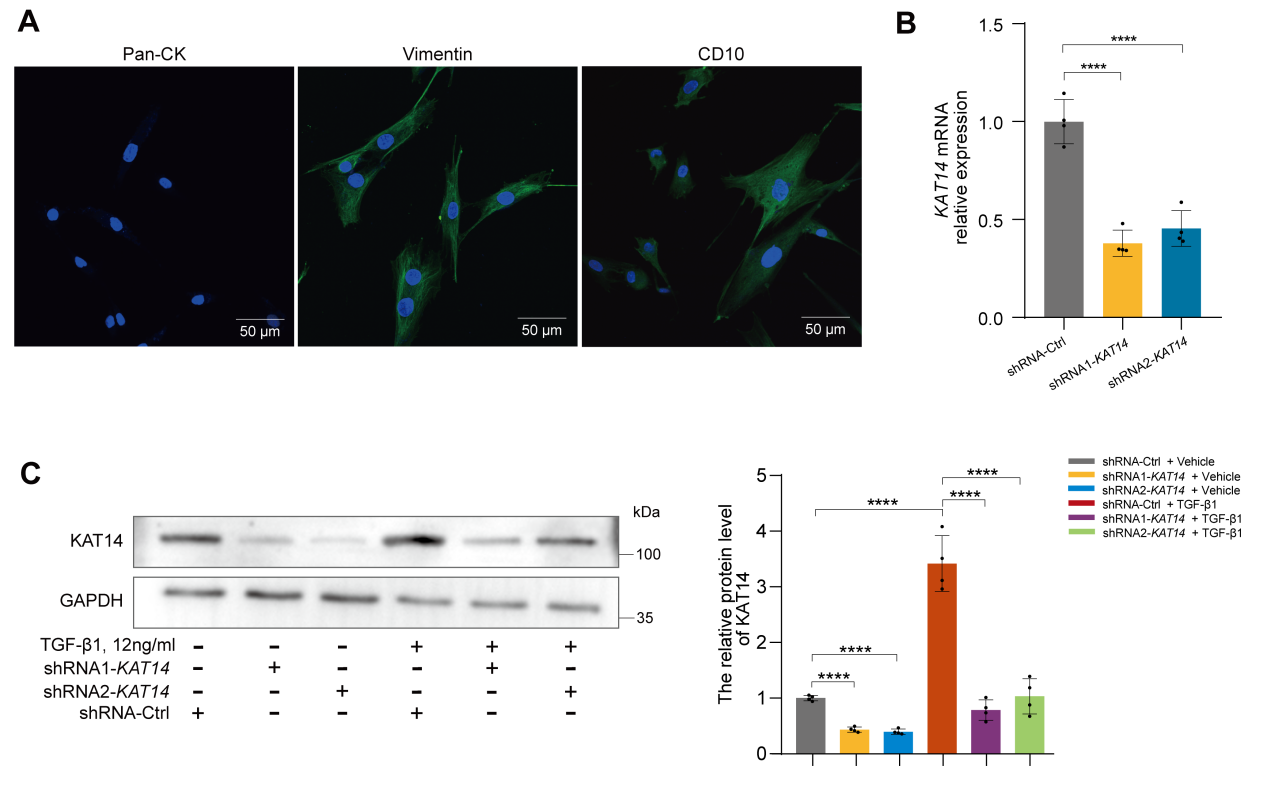


**Figure S6**. **Expression of KAT14 was significantly decreased in primary EcESCs treated with TGF-β1 following transfection with shRNA-*KAT14***. (**A**) Immunofluorescence staining showing the Pan-CK (endothelial cells marker), vimentin (a marker of stromal cells) and CD10 (a marker of endometrial stromal cells) in isolated primary endometriotic stromal cells. Scale bar: 50 μm. (**B**) qRT-PCR analysis of the relative mRNA expression of *KAT14* in primary EcESCs infected with lentiviral vector containing shRNA1-*KAT14*, shRNA2-*KAT14,* or shRNA-ctrl. Relative quantification of gene expression was calculated using the 2^−△△Ct^ method and normalized to *GAPDH* as the internal control. (**C**) Western blots measuring the protein level of KAT14 in primary EcESCs infected with shRNA1-*KAT14*, shRNA2-*KAT14,* or shRNA-ctrl lentiviruses stimulated by 12 ng/ml TGF-β1 for 24 h. Data are representative of three or more independent experimental replicates. For all panels, data are presented as the mean ± SD. *P*-values were determined by Student’s t-test in panel (**C**), and by one-way ANOVA in panels (**B**, **C**). *****P* < 0.0001, Ctrl: control, EcESCs: ectopic endometrial stromal cells, EuESCs: eutopic endometrial stromal cells, NESCs: normal endometrial stromal cells.


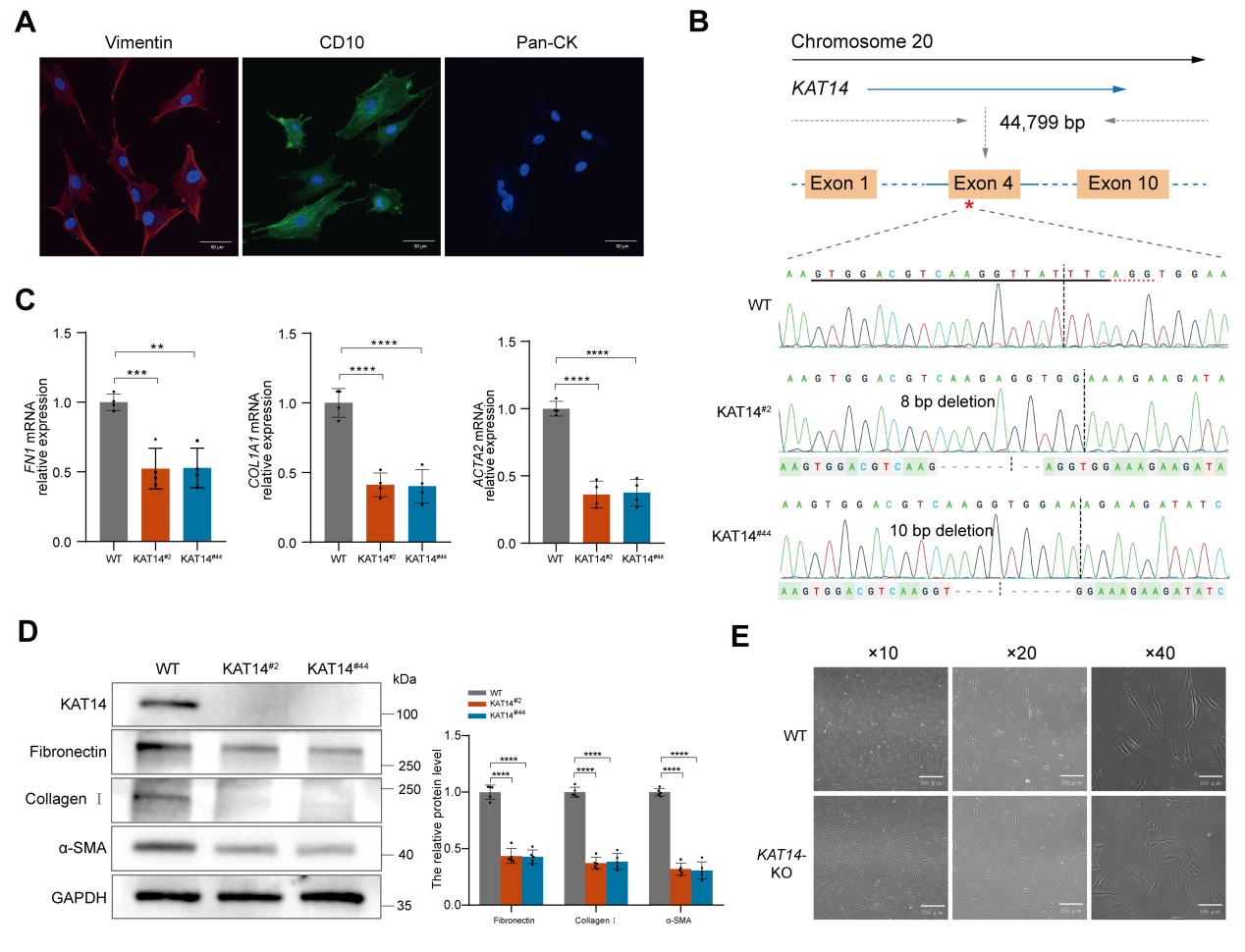


**Figure S7. Construction of *KAT14* knockout immortalized HESCs via the CRISPR/Cas9 technique.** (**A**) Immunofluorescence staining showing Pan-CK (endothelial cells marker), vimentin (a marker of stromal cells), and CD10 (a marker of endometrial stromal cells) in HESCs. Scale bar: 50 μm. (**B**) Schematic diagram of *KAT14* and the CRISPR/Cas9 vector target-site position. The target sites was designed based on the sequence at exon 4 (upper panel). Alignment of DNA sequences between wild-type and KAT14^#2^ HESCs showed a homozygous deletion (8-bp), and KAT14^#44^ HESCs showed a 10-bp homozygous deletion compared to wild-type (lower panel). (**C**) qRT-PCR showed the relative expression of *KAT14*, *FN1*, *COL1A1*, and *ACTA2* in *KAT14*-KO HESCs and wild-type cells. (**D**) Western blot assay showed the protein level of KAT14, fibronectin, collagen I, and α-SMA in *KAT14*-KO HESCs and wild-type cells. (**E**) Impact of *KAT14* KO on the morphology of HESCs. Scale bar: 500 μm (left panel), 200 μm (middle panel), and 100 μm (right panel). Relative quantification of gene expression was calculated using the 2^−△△Ct^ method and normalized to *GAPDH* as the internal control. Data are representative of three or more independent experimental replicates. For all panels, data are presented as the mean ± SD. *P*-values were determined by Student’s t-test. ***P* < 0.01, ****P* < 0.001, *****P* < 0.0001, HESCs: human endometrial stromal cells, KO: knockout, WT: wild-type.


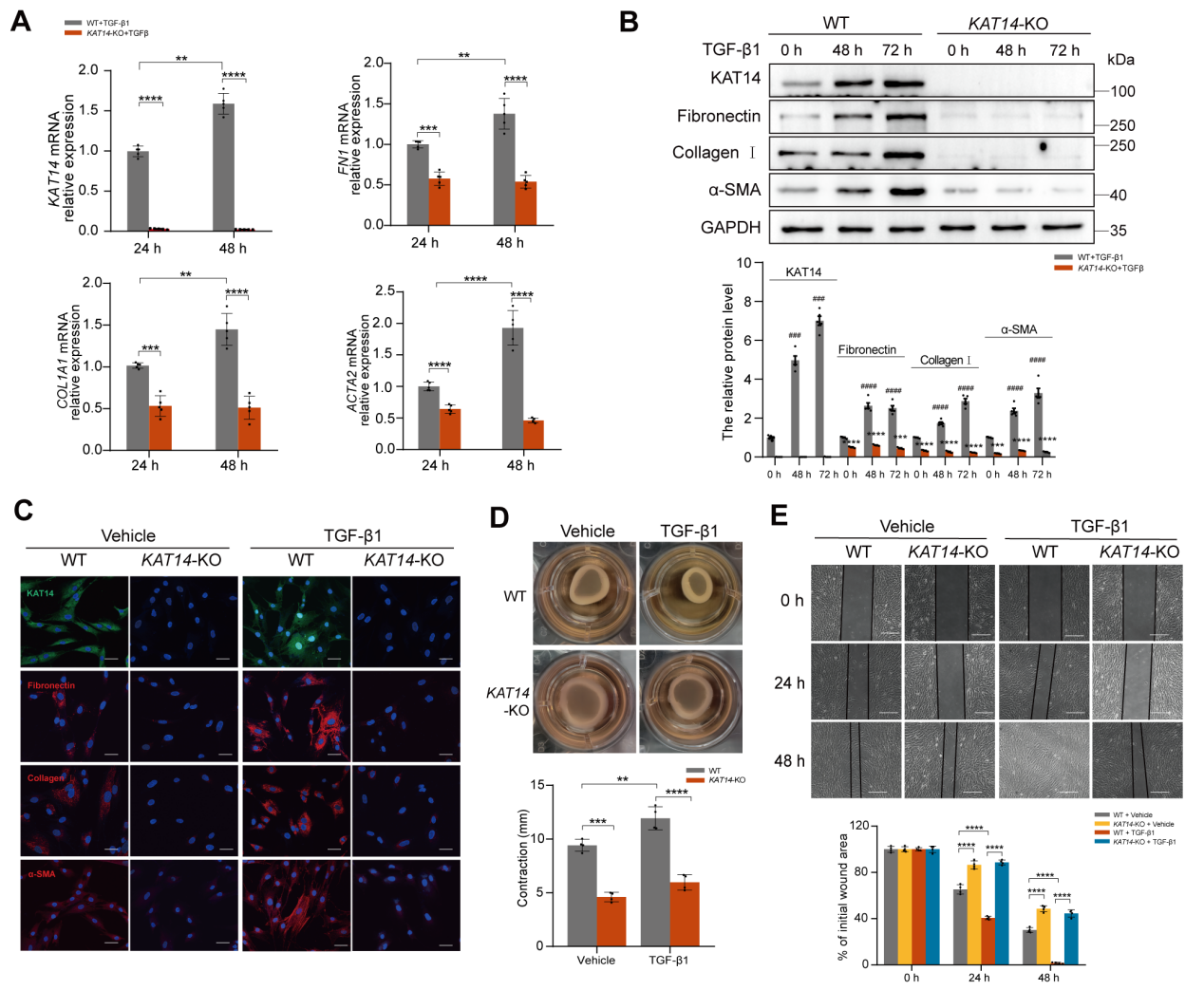


**Figure S8. *KAT14* knockout abrogated TGF-β1-induced fibrogenesis in immortalized HESCs.** (**A**) qRT-PCR analysis of the relative mRNA expression of *KAT14*, *FN1*, *COL1A1*, and *ACTA2* in *KAT14*-KO HESCs and wild-type cells treated with TGF-β1 or vehicle for 24 h and 48 h. Relative quantification of gene expression was calculated using the 2^−△△Ct^ method and normalized to *GAPDH* as the internal control. (**B**) Western blots measuring the protein level of KAT14, fibronectin, collagen I, and α-SMA in *KAT14*-KO HESCs and wild-type cells stimulated by TGF-β1 or vehicle for 0 h, 48 h, and 72 h. ****P* < 0.001 and *****P* < 0.0001 *KAT14*-KO versus wild-type HESCs; ^###^*P* < 0.001, ^####^*P* < 0.0001 wild-type HESCs at 48 h or 72 h versus wild-type HESCs at 0 h. (**C**) Immunofluorescence staining showing the expression of KAT14, α-SMA, and the ECM molecules fibronectin and collagen I in *KAT14*-KO HESCs and wild-type cells driven by TGF-β1 treatment or vehicle for 48 h. Scale bar: 50 μm. (**D**) Collagen gel contractility assay showing the cell contraction capacity of *KAT14*-KO HESCs and wild-type cells treated with TGF-β1 or vehicle for 24 h. The degree of collagen gel contraction was determined as the difference between the diameters of the well and the released gels. (**E**) Wound healing assay showing the migration ability of *KAT14*-KO HESCs and wild-type cells treated with TGF-β1 or vehicle for 0, 24, and 48 h. Wound healing was assessed by calculating the area in μm^2^ between the lesion edges. Scale bar: 200 μm. The concentration of TGF-β1 used for panels (**A**–**E**) was 12 ng/ml. Data are representative of three or more independent experimental replicates. For all panels, data are presented as the mean ± SD. *P*-values were determined by Student’s t-test in panels (**A**, **B**, **D**), and by one-way ANOVA in panels (**B**, **E**). ***P* < 0.01, ****P* < 0.001, *****P* < 0.0001, HESCs: human endometrial stromal cells, KO: knockout, WT: wild-type.


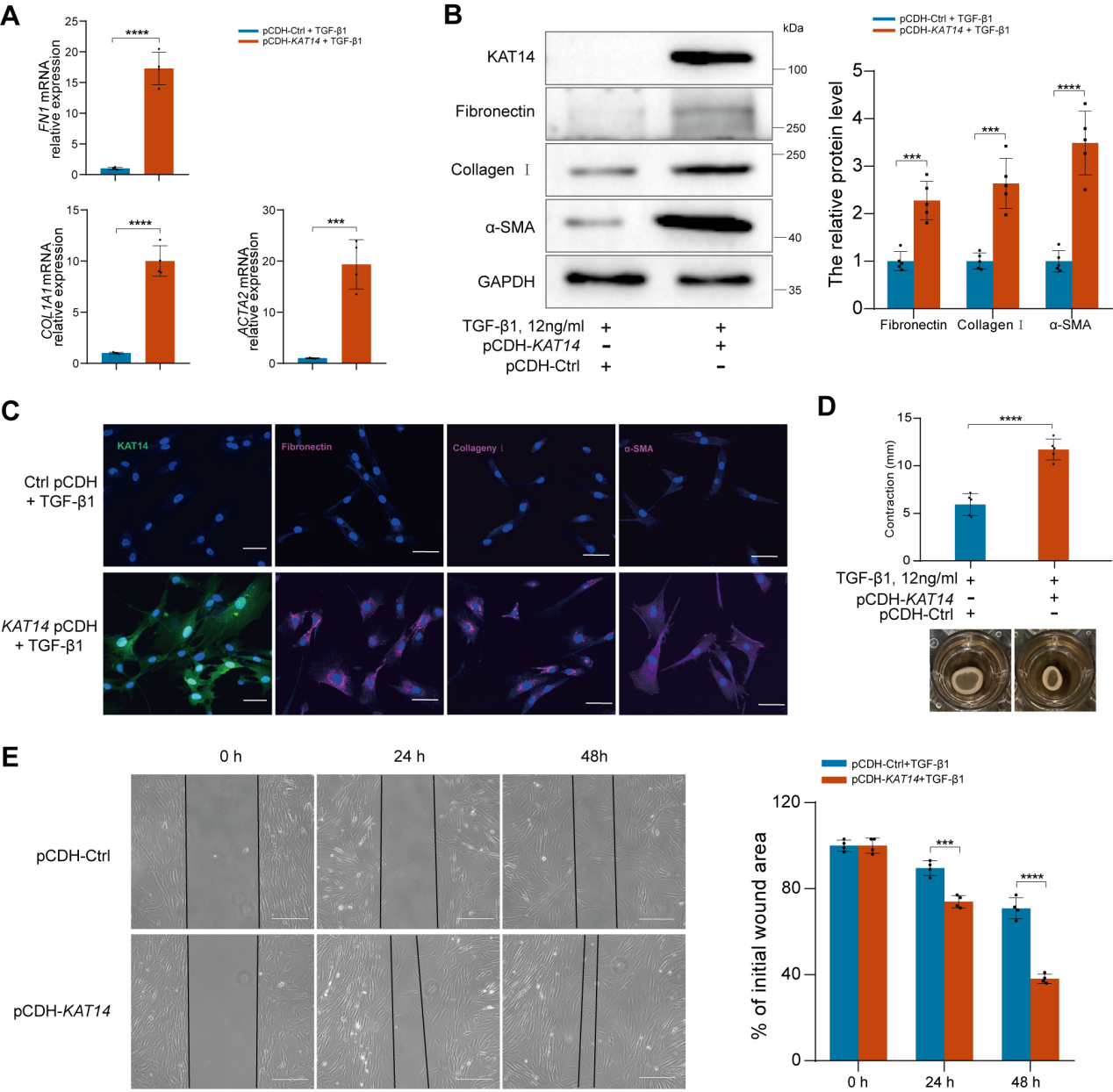


**Figure S9. Re-enhancement of *KAT14* reversed TGF-β1-induced fibrogenesis in *KAT14*-knockout HESCs.** (**A**) Results of qRT-PCR showing the relative mRNA expression of *FN1*, *COL1A1*, and *ACTA2* in *KAT14*-KO HESCs transfected with the indicated lentiviruses harboring *KAT14* expression vector (pCDH-*KAT14*) or empty vector control (pCDH-Ctrl) under TGF-β1 stimulation for 24 h. Relative quantification of gene expression was calculated using the 2^−△△Ct^ method and normalized to *GAPDH* as the internal control. (**B**) Western blots showing KAT14, fibronectin, collagen I, and α-SMA protein expression in *KAT14*-KO HESCs infected with pCDH-*KAT14* or pCDH-Ctrl lentiviruses under TGF-β1 stimulation for 48 h. (**C**) Immunofluorescence staining of α-SMA, and the ECM molecules fibronectin and collagen I in *KAT14*-KO HESCs infected with pCDH-*KAT14* or pCDH-Ctrl lentiviruses under TGF-β1 stimulation for 48 h. Scale bar: 50 μm. (**D**) Results of the collagen gel contractility assay showing the cell contraction capacity of *KAT14*-KO HESCs infected with pCDH-*KAT14* or pCDH-Ctrl lentiviruses under TGF-β1 stimulation for 24 h. (**E**) Migratory capacity of *KAT14*-KO HESCs infected with pCDH-*KAT14* or pCDH-Ctrl lentiviruses was measured by wound healing assay under TGF-β1 stimulation for 0, 24 and 48 h. Scale bar: 200 μm. The concentration of TGF-β1 used for panels (**A**–**E**) was 12 ng/ml. Data are representative of three or more independent experimental replicates. For all panels, data are presented as the mean ± SD. *P*-values were determined by Student’s t-test. ****P* < 0.001, *****P* < 0.0001, Ctrl: control, HESCs: human endometrial stromal cells, KO: knockout, WT: wild-type.


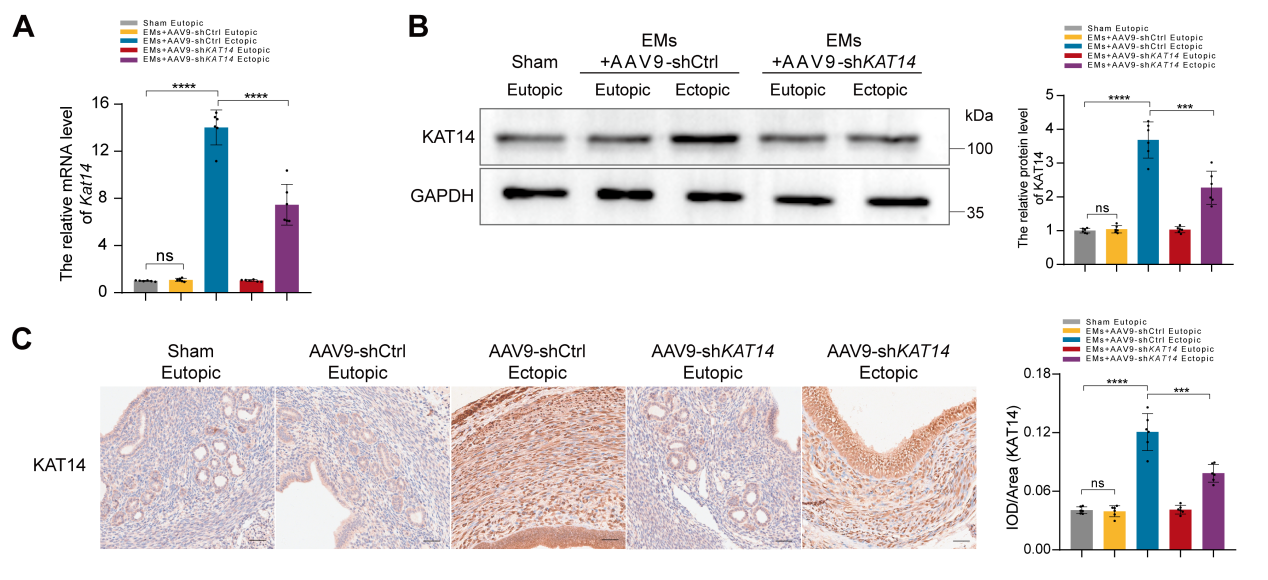


**Figure S10. Aberrantly upregulated KAT14 in ectopic lesions from endometriosis mice.** (**A**) RT-qPCR analysis of *Kat14* mRNA expression in endometrial tissues from mice in the sham group, eutopic endometrial tissues, and ectopic lesions from endometriosis mice injected with AAV9-shCtrl or AAV9-sh*KAT14*. Relative quantification of gene expression was calculated using the 2^−△△Ct^ method and normalized to *Gapdh* as the internal control; n = 6 mice per group. (**B**) Western blot analysis of KAT14 protein expression in endometrial tissues from mice in the sham group, eutopic endometrial tissues, and ectopic lesions from endometriosis mice injected with AAV9-shCtrl or AAV9-sh*KAT14*; n = 6 mice per group. (**C**) Representative images of KAT14 immunohistochemistry staining in endometrial tissues from mice in the sham group, eutopic endometrial tissues, and ectopic lesions from endometriosis mice injected with AAV9-shCtrl or AAV9-sh*KAT14*; n = 6 mice per group. Scale bar: 50 μm. Data are representative of three or more independent experimental replicates. For all panels, data are presented as the mean ± SD. *P*-values were determined through one-way ANOVA test. ****P* < 0.001, *****P* < 0.0001, ns: Not significant, AAV: adeno-associated virus, EMs: endometriomas.


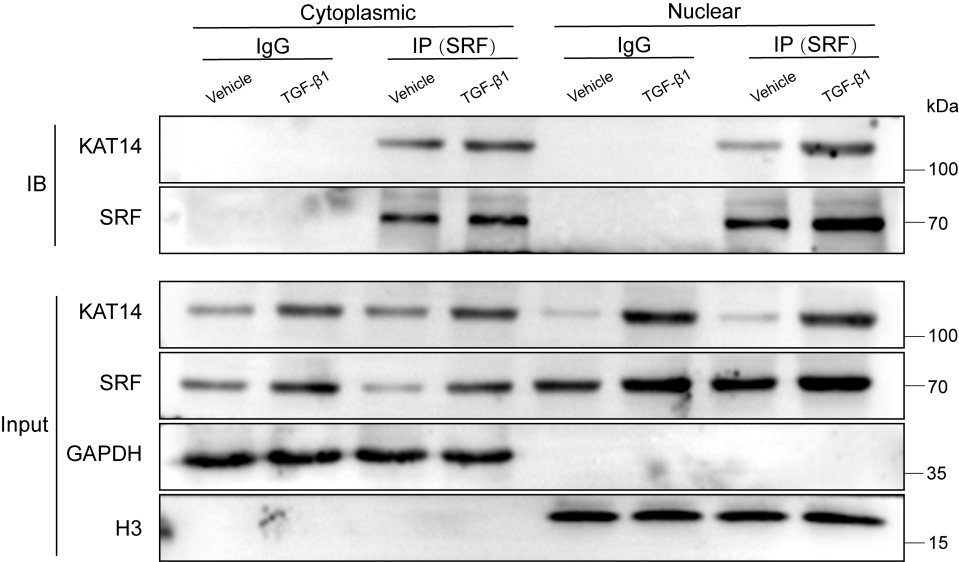


**Figure S11.** **TGF-β1 promoted KAT14 translocation into the nucleus via interacting with SRF in primary EcESCs.** Immunoprecipitation of SRF from the cytoplasm and nucleus fraction isolated from primary EcESCs treated with or without 12 ng/ml TGF-β1 confirms a signal for KAT14 co-immunoprecipitation. The purity of the cytoplasmic and nuclear fractions was confirmed by GAPDH and Histone-H3 antibodies. Data are representative of three or more independent experimental replicates. EcESCs: ectopic endometrial stromal cells.


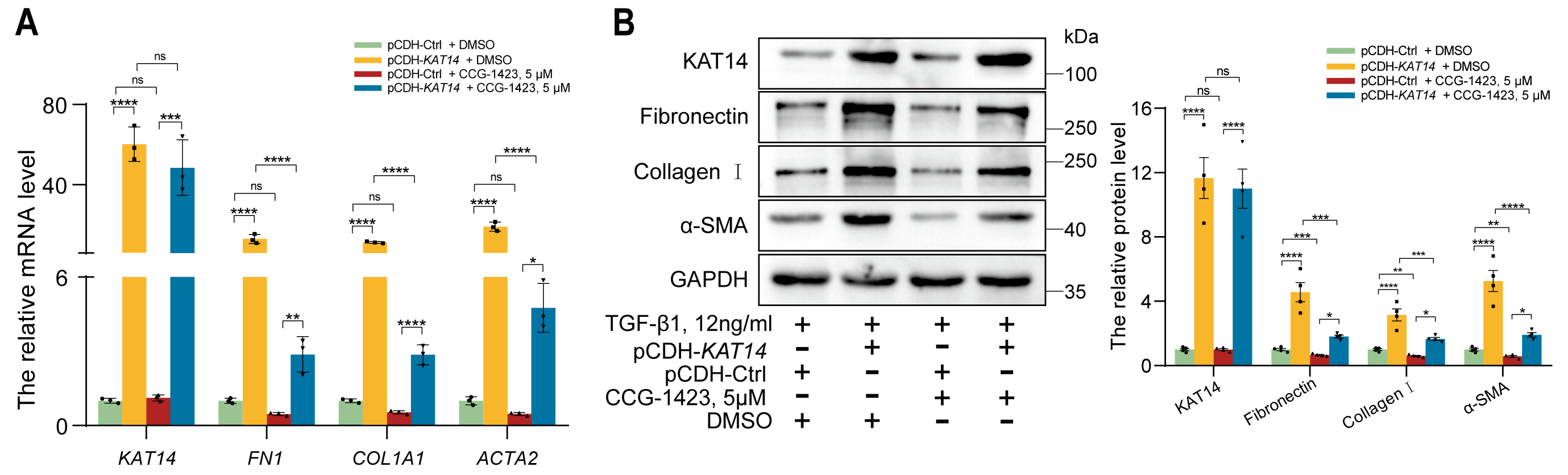


**Figure S12.** **SRF inhibitor CCG-1423 attenuated KAT14 re-expression-induced EcESC activation and ECM production under TGF-β1**. (**A**) qRT-PCR analysis showing the mRNA levels of *KAT14, FN1*, *COL1A1*, and *ACTA2* in *KAT14*-KD primary EcESCs infected with pCDH-*KAT14* or pCDH-Ctrl lentiviruses in the presence of CCG-1423 or DMSO stimulated with TGF-β1 for 24 h. Relative quantification of gene expression was calculated using the 2^−△△Ct^ method and normalized to *GAPDH* as the internal control. (**B**) Immunoblotting analysis showing the protein levels of KAT14, fibronectin, collagen 1, and α-SMA in *KAT14*-KD primary EcESCs transfected with lentiviruses harboring pCDH-*KAT14* or pCDH-Ctrl vectors in the presence of CCG-1423 or DMSO stimulated with TGF-β1 for 48 h. For stimulation, CCG-1423 was used at a concentration of 5 μM. Data are representative of three or more independent experimental replicates. For all panels, data are presented as the mean ± SD. *P*-values were determined by Student’s t-test. **P* < 0.05, ***P* < 0.01, ****P* < 0.001, *****P* < 0.0001, ns: Not significant, Ctrl: control, EcESCs: ectopic endometrial stromal cells, KD: knockdown.

**Additional Tables**

**Table S1. Phenotypic characteristics of normal control and ovarian endometriosis patients.** Related to Materials and Methods.

| **Group** | **Patient ID** | **Age (year)** | **BMI (kg/m^2^)** | **History of gestation** | **r-AFS stage** | **Other diagnosis** | **Menstrual average cycle (day)** | **Menstrual duration cycle (day)** | **CA125 (U/ml)** |
| --- | --- | --- | --- | --- | --- | --- | --- | --- | --- |
| **Ovarian endometriosis (OE) patients** | OE1 | 23 | 22 | G0P0 | III | None | 31 | 6 | 25.60 |
|  | OE2 | 31 | 21 | G2P1 | IV | None | 33 | 5 | 50.70 |
|  | OE3 | 28 | 21 | G2P1 | IV | None | 28 | 5 | 50.70 |
|  | OE4 | 32 | 18 | G3P1 | III | None | 31 | 5 | 24.30 |
|  | OE5 | 37 | 20 | G1P1 | IV | None | 30 | 4 | 86.60 |
|  | OE6 | 30 | 19 | G0P0 | III | None | 26 | 6 | 43.80 |
|  | OE7 | 38 | 23 | G2P1 | IV | None | 31 | 6 | 140.60 |
|  | OE8 | 27 | 22 | G2P0 | III | None | 35 | 5 | 73.57 |
|  | OE9 | 33 | 21 | G5P3 | IV | None | 40 | 5 | 165.40 |
|  | OE10 | 31 | 16 | G2P1 | IV | None | 30 | 6 | 61.70 |
|  | OE11 | 35 | 24 | G3P2 | III | Hydrosalpinx | 29 | 7 | 71.86 |
|  | OE12 | 40 | 18 | G2P1 | IV | None | 31 | 6 | 44.80 |
|  | OE13 | 29 | 21 | G0P0 | III | None | 30 | 5 | 21.40 |
|  | OE14 | 33 | 18 | G4P1 | III | Myoma | 28 | 5 | 40.20 |
|  | OE15 | 31 | 21 | G0P0 | IV | None | 28 | 6 | 52.90 |
|  | OE16 | 38 | 19 | G3P2 | III | None | 27 | 5 | 46.20 |
|  | OE17 | 28 | 22 | G0P0 | IV | None | 31 | 6 | 30.50 |
|  | OE18 | 35 | 21 | G1P1 | III | None | 30 | 7 | 181.90 |
|  | OE19 | 33 | 24 | G1P1 | III | None | 33 | 5 | 84.80 |
|  | OE20 | 26 | 20 | G4P0 | III | None | 38 | 5 | 39.50 |
|  | OE21 | 26 | 19 | G1P0 | III | Mesosalpinx cyst | 31 | 4 | 40.50 |
|  | OE22 | 31 | 21 | G3P1 | IV | DIE | 35 | 6 | 39.93 |
|  | OE23 | 33 | 21 | G0P0 | III | Myoma | 32 | 7 | 56.10 |
|  | OE24 | 32 | 23 | G2P0 | III | Ovarian simple cyst | 31 | 5 | 68.50 |
|  | OE25 | 36 | 19 | G2P1 | IV | None | 29 | 5 | 77.30 |
|  | OE26 | 29 | 21 | G2P0 | III | Myoma | 28 | 5 | 60.60 |
|  | OE27 | 33 | 23 | G1P0 | III | None | 33 | 6 | 74.20 |
|  | OE28 | 38 | 20 | G3P1 | III | None | 30 | 7 | 101.60 |
|  | OE29 | 35 | 22 | G1P1 | IV | None | 31 | 6 | 87.70 |
|  | OE30 | 29 | 21 | G0P1 | III | None | 32 | 7 | 79.10 |
|  | OE31 | 31 | 23 | G1P0 | III | Ovarian simple cyst | 31 | 5 | 53.90 |
| **Normal Control** | Ctrl1 | 35 | 24 | G3P2 | None | tubal infertility | 29 | 7 | 19.96 |
|  | Ctrl2 | 38 | 26 | G0P0 | None | uterine myomas | 35 | 5 | 21.86 |
|  | Ctrl3 | 37 | 20 | G1P0 | None | uterine myomas | 31 | 8 | 21.43 |
|  | Ctrl4 | 32 | 21 | G0P0 | None | uterine myomas | 33 | 6 | 22.97 |
|  | Ctrl5 | 27 | 22 | G1P0 | None | tubal infertility | 30 | 5 | 24.80 |
|  | Ctrl6 | 33 | 19 | G0P0 | None | tubal infertility | 28 | 7 | 29.71 |
|  | Ctrl7 | 35 | 18 | G3P0 | None | tubal infertility | 29 | 5 | 19.72 |
|  | Ctrl8 | 32 | 27 | G2P2 | None | uterine myomas | 31 | 7 | 17.57 |
|  | Ctrl9 | 30 | 20 | G0P0 | None | tubal infertility | 32 | 5 | 14.36 |
|  | Ctrl10 | 35 | 21 | G1P1 | None | uterine myomas | 33 | 5 | 20.50 |
|  | Ctrl11 | 33 | 20 | G0P0 | None | tubal infertility | 30 | 6 | 11.90 |
|  | Ctrl12 | 33 | 25 | G0P0 | None | tubal infertility | 29 | 5 | 13.80 |
|  | Ctrl13 | 37 | 19 | G1P1 | None | uterine myomas | 28 | 7 | 22.01 |
|  | Ctrl14 | 32 | 18 | G1P1 | None | uterine myomas | 27 | 8 | 17.78 |
|  | Ctrl15 | 39 | 19 | G1P1 | None | tubal infertility | 31 | 7 | 25.80 |
|  | Ctrl16 | 36 | 25 | G2P0 | None | uterine myomas | 32 | 6 | 30.30 |
|  | Ctrl17 | 34 | 20 | G0P0 | None | tubal infertility | 34 | 5 | 16.70 |
|  | Ctrl18 | 37 | 20 | G5P3 | None | uterine myomas | 30 | 5 | 21.00 |
|  | Ctrl19 | 26 | 18 | G0P0 | None | tubal infertility | 30 | 6 | 25.26 |
|  | Ctrl20 | 32 | 17 | G0P0 | None | uterine myomas | 31 | 5 | 17.77 |
|  | Ctrl21 | 31 | 22 | G0P0 | None | uterine myomas | 28 | 7 | 19.61 |
|  | Ctrl22 | 32 | 21 | G0P0 | None | tubal infertility | 26 | 5 | 24.30 |
|  | Ctrl23 | 30 | 26 | G1P0 | None | tubal infertility | 33 | 6 | 21.92 |

**Table S2. Phenotypic characteristics of normal control and OE patients.** Related to Materials and Methods.

| **Characteristics** | **OE patients ( n = 31 )** | **Normal Control ( n = 23 )** | ***p* value** |
| --- | --- | --- | --- |
| Age (year) | 32.0 ± 4.0 | 33.3 ± 3.3 | 0.19^a^ |
| BMI (kg/m^2^) | 20.8 ± 1.9 | 21.2 ± 2.9 | 0.95^b^ |
| CA125 level (U/ml) | 67.0 ± 38.0 | 20.9 ± 4.6 | < 0.0001^a^ |
| Menstrual average cycle (day) | 31.1 ± 3.0 | 30.4 ± 2.3 | 0.38^a^ |
| Menstrual duration cycle (day) | 5.6 ± 0.8 | 6.0 ± 1.0 | 0.12^a^ |
| r-AFS stage |  |  |  |
| Stage Ⅰ (minimal) | 0/31 |  |  |
| Stage Ⅱ (mild) | 0/31 |  |  |
| Stage Ⅲ (moderate) | 19/31 |  |  |
| Stage Ⅳ (severe) | 12/31 |  |  |
| Variables are expressed as mean ± SD. a: Estimated by Unpaired t test with Welch's correction. b: Estimated by Nonparametric test with Mann Whitney U test. OE, ovary endometriosis; rAFS scores, revised American Fertility Society Score; BMI, body mass index. | | | |

**Table S3. Sequences of sgRNA, shRNA and siRNA against specific target in this study.** Related to Materials and Methods.

| **shRNA&siRNA** |  | **Target sequence** |
| --- | --- | --- |
| Human *KAT14* sgRNA | 5’-3’ | GTGGACGTCAAGGTTATTTC |
| Human *KAT14* shRNA#1 | 5’-3’ | GGAAGGAAGTGGACGTCAAGG |
| Human *KAT14* shRNA#2 | 5’-3’ | GCCGCAGGCCAGATGTGATTC |
| Human *KAT14* shRNA#3 | 5’-3’ | GCCCTTATACCTCTCGGATCT |
| Human *SRF* siRNA#1 | 5’-3’ | GTGAGACAGGCCATGTGTA |
| Human *SRF* siRNA#2 | 5’-3’ | TGAGTGCCACTGGCTTTGA |
| Human *SRF* siRNA#3 | 5’-3’ | CTCAATTTGCTATGAGTATTA |
| Mouse *Kat14* shRNA#1 | 5’-3’ | CCAAAGTATTATCAGCCCTTA |
| Mouse *Kat14* shRNA#2 | 5’-3’ | GCTGTCTTACTTCTGTGACAA |
| Mouse *Kat14* shRNA#3 | 5’-3’ | CAAGTTTATTTCCGGTGGAAA |

**Table S4. Information of primers used in this study.** Related to Materials and Methods.

| Propose | Gene/Loci | Primer Sequences (5'→3') | |
| --- | --- | --- | --- |
|  |  | Forward | Reverse |
| cDNA cloning | Human KAT14#1 | GCAGAAGGGATGGATAGTAGCA | CTACTTGTCATCGTCATCCTTG |
|  | Human KAT14#2 | GCCACCATGGATAGTAGCAT | GGATCCTCAGGCATAATCTG |
|  | Human SRF | ATGCTGCCTACACAAGCTG | CGCGGATCCTCATTTGTCGTCA |
| Real-time qPCR | Human FN1 | GATAAATCAACAGTGGGAGC | CCCAGATCATGGAGTCTTTA |
|  | Human COL1A1 | CCTCCAGGGCTCCAACGAG | TCTATCACTGTCTTGCCCCA |
|  | Human KAT14 | CTTGACCGATACCAGACTTCC | CAGGTGGGAACGAATCTGTGA |
|  | Human SRF | TTCAGCTCCACCAGATGGC | GGCTCAGTCCTGTGGCTG |
|  | Human HDAC3 | ACCAATATGCAAGGCTTCACCAA | GCCTGTGTAACGCGAGCAGA |
|  | Human HDAC6 | AGTCCATCGCAGATACTGGC | TTAGTCTGGCCTGGAGTGGA |
|  | Human SIRT2 | CTGCGGAACTTATTCTCCCAGAC | CCACCAAACAGATGACTCTGCG |
|  | Human KAT5 | CCGCAAGGGCACCATCT | GGTAGGGAGGCAGGGTTAGG |
|  | Human KAT2A | TGGGAGTCAGGCTTCACCATGC | CACCCATGCTGGGGCTGAAG |
|  | Human TGFB1 | TGGTGGAAACCCACAACGAA | GAGCAACACGGGTTCAGGTA |
|  | Human ACTA2 | TGTATGTGGCTATCCAGGCG | AGAGTCCAGCACGATGCCAG |
|  | Human GAPDH | GTGAAGGTCGGAGTCAACG | TGAGGTCAATGAAGGGGTC |
|  | Mouse Fn1 | GGATCCCCTCCCAGAGAAGT | AGAGCTTCCTGTCCTGTCTTC |
|  | Mouse Col1a1 | GGAGAGAGCATGACCGATGG | AAGTTCCGGTGTGACTCGTG |
|  | Mouse Kat14 | GACCGGCAGAGGCTTCG | AACACACCACACCCACAGTT |
|  | Mouse Srf | CGCGTGAAGATCAAGATGGAG | GACAGCTCATAGGCCTTCTTC |
|  | Mouse Acta2 | CTTCGTGACTACTGCCGAGC | AGGTGGTTTCGTGGATGCC |
|  | Mouse Gapdh | CAGGAGAGTGTTTCCTCGTC | TGATGTTAGTGGGGTCTCGC |
| ChIP-qPCR | Human ACTA2 | AGTTTTGTGCTGAGGTCCCTATATG | TTCCCAAACAAGGAGCAAAGA |
|  | Human KPNA2 | TCCCTCCCATAGTAGCCAGA | GGCGACAGCCTTAAACAAAT |
| ChIP-PCR | Human ACTA2  promoter | GAGTTTTGTGCTGAGGTCCC | AGCTGGAGCTGCTTCACAG |
|  | Human GAPDH | TACTAGCGGTTTTACGGGCG | AGGAGGAGAGAGCGAA |
| **Knock out  screening PCR** | Human KAT14 | AATATTCCAAGATACCGTCC | CTGTAATCCCAGCTACTCA |

**Table S5. Details of antibodies.** Related to Materials and Methods.

| **Antibodies** | **Source** | **Catalogue number** | **RRID tag** | **Dilution in WB** | **Dilution in IHC** | **Dilution in IF** |
| --- | --- | --- | --- | --- | --- | --- |
| FN1 | Abcam | Cat#ab2413 | RRID:AB_2262874 | 1: 5000 | Not applied | 1:1000 |
| Collagen I | Abcam | Cat#ab138492 | RRID:AB_2861258 | 1: 1000 | Not applied | 1: 2000 |
| α-SMA#1 | Abcam | Cat#ab21027 | RRID:AB_1951138 | Not applied | Not applied | 1: 500 |
| α-SMA#2 | proteintech | Cat#14395-1-AP | RRID:AB_2223009 | 1: 1000 | 1: 2000 | 1: 2000 |
| SRF | proteintech | Cat#16821-1-AP | RRID:AB_2194384 | 1: 3000 | Not applied | 1: 200 |
| KAT14#1 | Santa Cruz  Biotechnology | Cat#sc-398475 | RRID:AB_2936359 | 1: 100 | Not applied | 1: 200 |
| KAT14#2 | Novus | Cat#NBP2-92912 | RRID:AB_2936360 | Not applied | 1: 100 | 1: 1000 |
| KPNA2 | proteintech | Cat#10819-1-AP | RRID:AB_2265526 |  |  |  |
| GAPDH | proteintech | Cat#60004-1-Ig | RRID:AB_2107436 | 1: 10000 | Not applied | Not applied |
| H3 | abcam | Cat#ab1791 | RRID:AB_302613 | 1: 2000 | Not applied | Not applied |
| H4 | abcam | Cat#ab9051 | RRID:AB_306967 | 1: 1000 | Not applied | Not applied |
| H3ac | proteintech | Cat#17168-1-AP | RRID:AB_2716755 | 1: 5000 | Not applied | Not applied |
| H4ac | proteintech | Cat#39043 | RRID:AB_2793201 | 1: 5000 | Not applied | Not applied |
| RNAPII | abcam | Cat#ab26721 | RRID:AB_777726 | Not applied | Not applied | Not applied |
| IgG | proteintech | Cat#10284-1-AP | RRID:AB_2877729 | 1: 10000 | Not applied | Not applied |
| vimentin#1 | abcam | Cat#ab92547 | RRID:AB_10562134 | Not applied | 1: 200 | Not applied |
| vimentin#2 | proteintech | Cat#60330-1-Ig | RRID:AB_2881439 | Not applied | Not applied | 1：1000 |
| pan-cytokeratin#1 | abcam | Cat#ab7753 | RRID:AB_306047 | Not applied | 1: 1000 | Not applied |
| pan-cytokeratin#2 | proteintech | Cat#26411-1-AP | RRID:AB_2880505 | Not applied | Not applied | 1：400 |
| MME, CD10 | proteintech | Cat#23898-1-AP | RRID:AB_2879354 | Not applied | Not applied | 1：200 |
| KAT2A/GCN5 | proteintech | Cat#66575-1 | RRID:AB_2881935 | 1:5000 | Not applied | Not applied |
| TIP60/KAT5 | proteintech | Cat#10827-1-AP | RRID:AB_2128431 | 1:2000 | Not applied | Not applied |
| SIRT2 | proteintech | Cat#19655-1-AP | RRID:AB_2878592 | 1:30000 | Not applied | Not applied |
| HDAC3 | proteintech | Cat#10255-1-AP | RRID:AB_2279733 | 1:2000 | Not applied | Not applied |
| HDAC6 | proteintech | Cat#12834-1-AP | RRID:AB_10597094 | 1:1000 | Not applied | Not applied |
| βTubulin | proteintech | Cat#10094-1-AP | RRID:AB_2210695 | 1: 2000 | Not applied | Not applied |
| Goat Anti-Mouse | proteintech | Cat#SA00001-1 | RRID:AB_2722565 | 1: 10000 | Not applied | Not applied |
| Goat Anti-Rabbit | proteintech | Cat#SA00001-2 | RRID:AB_2722564 | 1: 10000 | Not applied | Not applied |
| Donkey Anti-Goat | proteintech | Cat#SA00001-3 | RRID:AB_2890882 | 1: 10000 | Not applied | Not applied |
| Goat Anti-Mouse (Alexa Fluor® 488) | proteintech | Cat#SA00013-1 | RRID:AB_2810983 | Not applied | Not applied | 1: 500 |
| Donkey Anti-Rabbit (Alexa Fluor® 647) | abcam | Cat#ab150075 | RRID:AB_2752244 | Not applied | Not applied | 1: 500 |
| Donkey Anti-Goat (Alexa Fluor® 568) | abcam | Cat#ab175704 | RRID:AB_2725786 | Not applied | Not applied | 1: 500 |
| TGF Beta 1 | proteintech | Cat#21898-AP | RRID:AB_2811115 | 1: 3000 | 1: 100 | Not applied |
